# Degradation Signals for Ubiquitin-Proteasome Dependent Cytosolic Protein Quality Control (CytoQC) in Yeast

**DOI:** 10.1534/g3.116.027953

**Published:** 2016-04-26

**Authors:** Matthew J. Maurer, Eric D. Spear, Allen T. Yu, Evan J. Lee, Saba Shahzad, Susan Michaelis

**Affiliations:** Department of Cell Biology, The Johns Hopkins University School of Medicine, Baltimore, Maryland 21205

**Keywords:** proteostasis, ubiquitin proteasome system, protein quality control, E3 ligase Doa10, Ltn1

## Abstract

Cellular protein quality control (PQC) systems selectively target misfolded or otherwise aberrant proteins for degradation by the ubiquitin-proteasome system (UPS). How cells discern abnormal from normal proteins remains incompletely understood, but involves in part the recognition between ubiquitin E3 ligases and degradation signals (degrons) that are exposed in misfolded proteins. PQC is compartmentalized in the cell, and a great deal has been learned in recent years about ER-associated degradation (ERAD) and nuclear quality control. In contrast, a comprehensive view of cytosolic quality control (CytoQC) has yet to emerge, and will benefit from the development of a well-defined set of model substrates. In this study, we generated an isogenic “degron library” in *Saccharomyces cerevisiae* consisting of short sequences appended to the C-terminus of a reporter protein, Ura3. About half of these degron-containing proteins are substrates of the integral membrane E3 ligase Doa10, which also plays a pivotal role in ERAD and some nuclear protein degradation. Notably, some of our degron fusion proteins exhibit dependence on the E3 ligase Ltn1/Rkr1 for degradation, apparently by a mechanism distinct from its known role in ribosomal quality control of translationally paused proteins. Ubr1 and San1, E3 ligases involved in the recognition of some misfolded CytoQC substrates, are largely dispensable for the degradation of our degron-containing proteins. Interestingly, the Hsp70/Hsp40 chaperone/cochaperones Ssa1,2 and Ydj1, are required for the degradation of all constructs tested. Taken together, the comprehensive degron library presented here provides an important resource of isogenic substrates for testing candidate PQC components and identifying new ones.

Protein quality control (PQC) mechanisms are critical for maintaining cellular proteostasis by recognizing aberrant proteins and degrading them. Irreversibly misfolded proteins are selectively degraded by the ubiquitin-proteasome system (UPS) (Balch *et al.* 2008; Buchberger *et al.* 2010). Misfolded or aggregation-prone proteins are associated with numerous human diseases due to loss of function through degradation (*i.e.*, cystic fibrosis), or toxic gain of function (*i.e.*, accumulation of protein aggregates in Huntington’s and Parkinson’s diseases) (Hol and Scheper 2008). Thus, understanding how misfolded proteins are targeted for destruction, or specifically evade PQC mechanisms, is critical for developing therapeutic strategies for protein folding diseases.

The UPS ensures that misfolded proteins are committed to degradation through the activities of a ubiquitin activating enzyme (E1), ubiquitin conjugating enzymes (E2s), and ubiquitin ligases (E3s) that append poly-ubiquitin chains to substrates, which in turn directs them to the proteasome. Importantly, the E3 ubiquitin ligases are thought to serve as the specificity factors leading to ubiquitylation of misfolded proteins (Finley 2009; Finley *et al.* 2012; Pickart 2004; Deshaies and Joazeiro 2009). In addition, Hsp70 chaperones and Hsp40 cochaperones play a role in PQC by preventing substrate aggregation, or by providing a “bridge” between misfolded substrate and a specific E3 ligase (McClellan *et al.* 2005b; Nakatsukasa *et al.* 2008; Nishikawa *et al.* 2005). The code that pairs specific ubiquitin E3 ligases with particular degradation signals, or “degrons”, within aberrant proteins is not yet fully understood. The budding yeast *Saccharomyces cerevisiae* has been a useful model organism for beginning to define degron–E3 interactions (Finley *et al.* 2012; Guerriero *et al.* 2013; Ravid and Hochstrasser 2008; Ravid *et al.* 2006; Vembar and Brodsky 2008), and its utility for this goal is expanded in the present study.

PQC machinery exists in various cellular compartments, including the endoplasmic reticulum (ER), nucleus, and cytoplasm. Until recently, the ER-associated protein degradation (ERAD) pathway, whereby misfolded or unassembled membrane and secretory proteins are degraded, has garnered the most attention. In yeast, ERAD substrates are recognized by one of two integral membrane E3 ligases, either Doa10 or Hrd1 (Bays *et al.* 2001; Swanson *et al.* 2001; Kreft *et al.* 2006). The site of the misfolded lesion within an ERAD substrate appears to determine which E3 is involved, such that Doa10 acts on membrane proteins with cytoplasmic lesions, and Hrd1 ubiquitylates proteins with misfolded luminal lesions (Huyer *et al.* 2004; Vashist and Ng 2004). While it had been thought that aberrant transmembrane spans are exclusively ubiquitylated by Hrd1, recent evidence suggests that Doa10 can also recognize misfolded transmembrane spans in some substrates (Habeck *et al.* 2015). In any case, Doa10 and Hrd1 can account for the ubiquitylation and degradation of essentially all ERAD substrates in yeast (Ismail and Ng 2006).

Significant progress has also been made in recent years defining the mechanisms of nuclear PQC in yeast. The well-studied nuclear E3 ligase, San1, directly binds and targets many temperature-sensitive (Ts^–^) nuclear proteins or nuclear localization signal (NLS)-directed GFP-degron fusions through its N- and C-terminal unstructured regions (Gardner *et al.* 2005; Rosenbaum *et al.* 2011; Rosenbaum and Gardner 2011). Elegant studies from Gardner and coworkers demonstrated that San1 targets substrates with a high propensity to aggregate, such as those having hydrophobic stretches of at least five contiguous residues (Fredrickson *et al.* 2011, 2013; Fredrickson and Gardner 2012). The E3 ligase Doa10 resides not only in the ER membrane, but also in the inner nuclear membrane (INM), and is required for the degradation of several nuclear quality control substrates, including a mutant version of the kinetochore protein Ndc10, and proteins bearing the MATα2-derived degron *Deg1* (Deng and Hochstrasser 2006; Ravid *et al.* 2006; Furth *et al.* 2011). More recently, the Asi complex that contains the E3s Asi1 and Asi3, and is also in the INM, has been shown to ubiquitylate membrane proteins that inadvertently mislocalize from the ER to the INM, such as the sterol biosynthesis enzymes Erg11 and Nsg1 (Foresti *et al.* 2014; Khmelinskii *et al.* 2014). Together, the nuclear E3s San1, Doa10, and Asi1/3 play diverse but related roles in regulation, removing misfolded or aggregated proteins from the nucleus, and safe-guarding nuclear membrane integrity.

Interestingly, much less is known about cytoplasmic protein quality control (CytoQC) than about ERAD or nuclear quality control. The synthesis of nearly all cellular proteins takes place in the cytoplasm. Thus, cells must distinguish between fully synthesized but irreversibly misfolded proteins that should be targeted for degradation, and nascent polypeptides that are not yet fully folded and are still ribosome-associated. The E3 ligases implicated to date as major players in yeast CytoQC are Doa10, Ubr1, and San1 (Fredrickson and Gardner 2012). These E3s, and the substrates upon which they act, are discussed further below. In addition, a ribosomal quality control (RQC) complex has recently been described that contains the E3 ligase Ltn1 (formerly called Rkr1) along with the additional components Rqc1 and Rqc2/Tae2 (Brandman *et al.* 2012; Shen *et al.* 2015). Ltn1 ubiquitylates polypeptides that undergo translational pausing during aberrant synthesis of poly-basic peptides, such as poly-lysine from the poly(A) tail of a nonstop mRNA on the ribosome (Bengtson and Joazeiro 2010). Such stalled proteins are cotranslationally ubiquitylated by Ltn1, targeting them for degradation even before their synthesis is complete.

It was surprising when studies by us and others revealed that the E3 ligase Doa10 is also involved in the degradation of several soluble CytoQC substrates (Metzger *et al.* 2008; Ravid *et al.* 2006), given that Doa10 is membrane-associated and clearly implicated in ERAD. These substrates include the mutant Ts^–^ cytosolic proteins Ura3-2 and Ura3-3, and the cytosolic reporter proteins Ura3-HA-CL1 and Ura3-GFP-CL1 that contain a C-terminal 16-amino-acid degron called CL1 (Lewis and Pelham 2009; Metzger *et al.* 2008). The finding that Doa10 is responsible for ubiquitylation of these model CytoQC substrates, in addition to its known role in ERAD, suggests that the cytoplasmic face of the ER membrane may be a common platform for CytoQC of numerous substrates, an issue we address in the present study.

Ubr1 is an E3 ligase first identified by its ability to target proteins with destabilizing N-terminal amino acids (N-end rule substrates) (Varshavsky 1997; Varshavsky *et al.* 2000). More recently, Ubr1 was shown to be critical for the degradation of immature protein kinases, mutant forms of cytosolic enzymes, as well as a number of engineered CytoQC substrates, including cytosolically localized mutant versions of GFP, proteinase A, carboxypeptidase Y and the ABC transporter Ste6 (Eisele and Wolf 2008; Guerriero *et al.* 2013; Heck *et al.* 2010; Nillegoda *et al.* 2010; Park *et al.* 2013; Prasad *et al.* 2010, 2012; Khosrow-Khavar *et al.* 2012; Summers *et al.* 2013). Interestingly, deletion of *SAN1* also stabilized some of these CytoQC substrates to varying extents, providing evidence that Ubr1 and San1 often act in a redundant fashion in CytoQC (Fredrickson and Gardner 2012). Surprisingly some of these CytoQC substrates are transported to the nucleus, explaining why nuclear San1 may be involved (Park *et al.* 2013; Prasad *et al.* 2010). However, others clearly remain in the cytoplasm, suggesting a role for San1 outside the nucleus (Guerriero *et al.* 2013). Alternatively, stabilized substrates in these cells could be exported out of the nucleus at a faster rate than nuclear import. Clearly, the basis for the interrelated but distinct preference of the Ubr1 and San1 E3 ligases remains incompletely understood.

Despite the growing list of CytoQC substrates, it has been difficult to identify characteristics within misfolded proteins that are recognized by the different E3 ligases in the cytoplasm. Thus, a comprehensive and unified view of CytoQC has yet to emerge and could benefit from development of a defined set of model substrates. In this study, we have generated an isogenic Ura3-degron fusion library to begin to systematically address the issue of the “code” for CytoQC. Within this collection of 77 degron-containing proteins, about half are substrates for Doa10, while the remaining ones are degraded independently of Doa10. The degradation of all of these is dependent on the proteasome, and in a more detailed analysis of a subset of these, all show dependence on the Hsp70 chaperones Ssa1/2 and Hsp40 Ydj1. Notably, while only a few of the degron library constructs are spared when Ubr1 or San1 are absent, and only modestly at that, a number of the degron constructs exhibit Ltn1-dependent degradation. We provide evidence that this may occur via a mechanism distinct from ribosomal pausing. Taken together, the degron library presented here will provide a valuable resource of defined substrates for future studies of CytoQC.

## Materials and Methods

### Yeast strains, media, and growth conditions

The *S. cerevisiae* strains used in this study are listed in Supplemental Material, Table S1. Standard media (SC-Leu and SC-Leu-Ura) (Michaelis and Herskowitz, 1988), and media used for proteasome inhibition with MG132 (Liu *et al.* 2007), are described in File S1. Unless otherwise indicated, yeast was grown at 30°. Yeast cultures in mid-log phase were obtained by dilution of overnight saturated cultures to an OD_600_ of 0.3, and growth for 3–4 hr, at which point an OD_600_ measurement was taken to confirm cultures were in mid-log phase (OD_600_ between 0.6 and 0.8). For spot growth tests, overnight saturated cultures were adjusted to 0.2 OD_600_/ml, and then 10-fold serial dilutions were spotted onto SC-Leu and SC-Leu-Ura plates and incubated for 2 days at 30°.

### Plasmids and plasmids constructions

**Figure 1:**
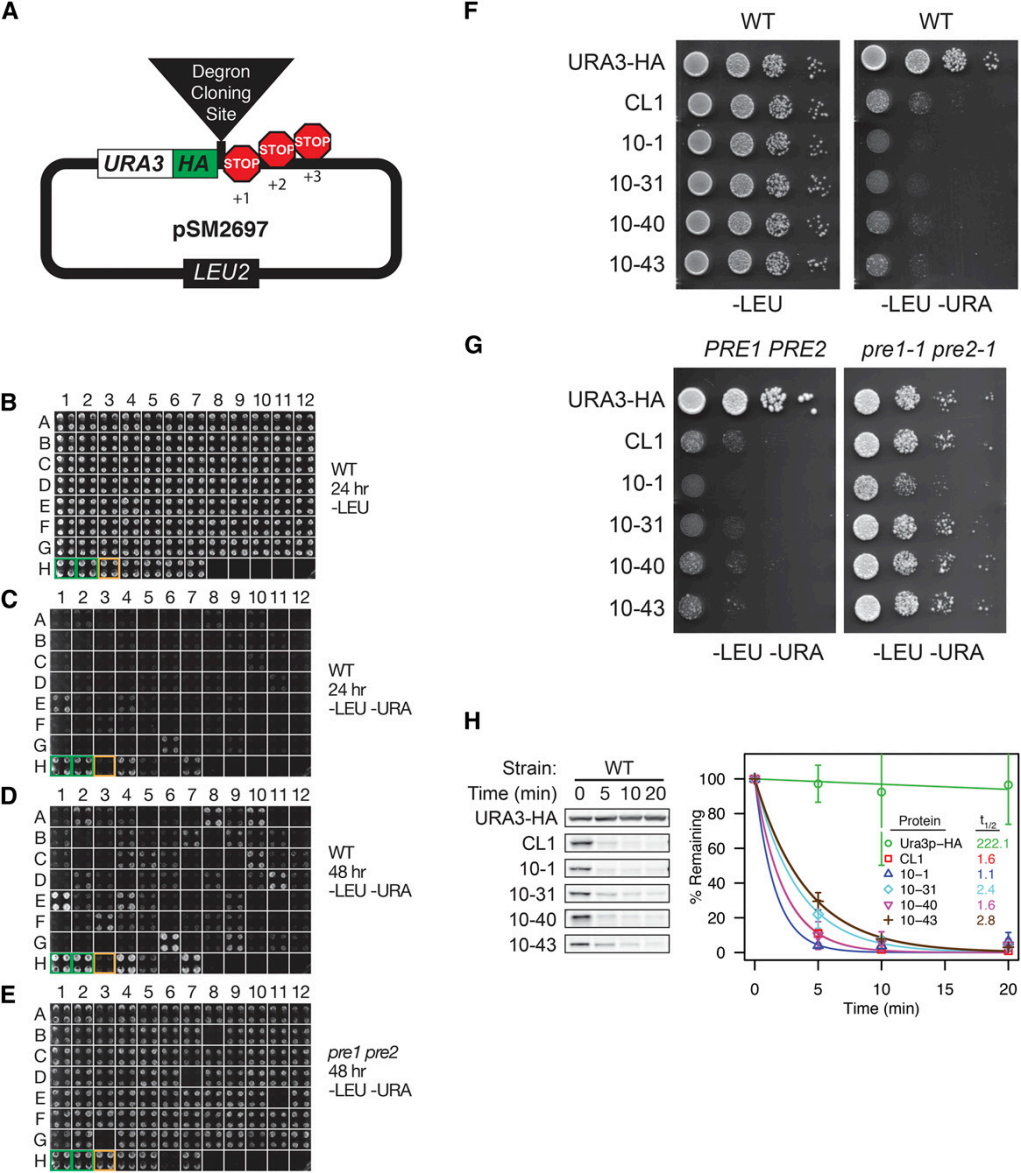
A genetic screen to isolate proteasome-dependent protein degradation signals (degrons). (A) Schematic of the library cloning vector. Yeast genomic DNA fragments were cloned into the library vector such that they would be expressed as a C-terminal fusion to Ura3-HA, followed by stop codons in all three frames. (B–E) Candidate clones isolated in the screen based on conferring a Ura– phenotype were arrayed in 96-well format, and retransformed into WT (SM4333) and proteasome mutant (*pre1 pre2*; SM4334) strains. Growth assays for 84 of the 153 isolates are shown in (B–D). Transformants were transferred from liquid growth medium onto the indicated agar plates, and incubated for the times shown. Controls expressing Ura3-HA (green boxes) and Ura3-HA-CL1 (orange box) were present in several positions on the plates. Additional degron-containing constructs generated in a previous study (Metzger *et al.* 2008) are also present on these plates (positions H4–H7: Tom20-Ura3-HA, Tom20-Ura3-HA-CL1, Vma12-Ura3-HA-CL1, and Vma12-Ura3-HA). (F–G) Spot dilution growth assay for several Ura3-HA-degron constructs from the library. Serial dilutions of WT yeast (SM4460) expressing Ura3-HA with no degron, Ura3-HA-CL1, and four Ura3-HA degron constructs were spotted to plates and incubated 48 hr (F). These constructs were also examined for growth in the proteasome mutant (SM4334; *pre1-1 pre2-1*) and isogenic WT strain (SM4333; *PRE1 PRE2*) to determine if growth improves when proteasome function is defective (G). (H) Ura3-HA-degron turnover was analyzed by cycloheximide chase analysis as described in *Materials and Methods*. A first-order exponential decay function was fit to each chase series, allowing determination of the half-lives (*t*_1/2_) for each degron-containing protein. The mean and 1 SD for three independent experiments are shown.

Plasmids used in this study are listed in Table S2. To generate the starting plasmid pSM2697 for the degron library (Figure 1A), recombinational cloning was used to add a fragment containing a *Sma*I site and three frame-shifted stop codons in place of the single stop codon in pSM2287 (Metzger *et al.* 2008). Plasmid pSM2697 is a *CEN LEU2* plasmid with *URA3-3HA* followed by the *Sma*I cloning site and stop codons in three frames. Construction of plasmids pSM2801, pSM2802, pSM2803, pSM2811, and pSM2812 is described in File S1.

### Degron library construction and screening

Yeast genomic DNA was digested with DNaseI in 50 mM Tris-HCl (pH 7.5), 5 mM MnCl_2_, 5 mM CaCl_2_ for various times and the reaction was quenched by the addition of 0.02 M EDTA (pH 8). A reaction yielding a distribution of DNA fragments ranging from ∼50 to 400 bp, as judged from electrophoresis analysis, was made blunt-ended with T4 DNA polymerase and cloned into the *Sma*I site of pSM2697. The ligation reaction was transformed into bacteria, and colonies from several plates (∼5000 colonies) were pooled, grown in LB plus carbenicillin, and plasmid DNA was purified to yield a library containing *Ura3-3XHA* with random 3′ inserts.

The degron plasmid library DNA (*CEN LEU2URA3-3XHA-insert*) was transformed into SM4460 (relevant genotype *leu2*∆*ura3*∆) and plated onto SC-Leu media to select for Leu^+^ yeast colonies containing individual library plasmids. Transformants were replica plated onto SC-Leu-Ura media to identify Ura^–^ clones. Of ∼3000 Leu+ colonies screened, 153 were Ura^–^ (∼5%). Plasmids prepared from these initial clones were passaged through *Escherichia. coli* to ensure each library clone represented a single plasmid. After DNA sequencing, 113 unique inserts were confirmed.

To determine proteasome-dependence of growth, yeast strains SM4333 (*ura3*∆ *leu2*∆) and SM4334 (*pre1-1 pre2-1 ura3*∆ *leu2*∆) were transformed (without heat shock), with the 113 individual library clones in two 96-deep-well plates, with controls on the bottom row. Transformants were grown to saturation in liquid culture, and then pinned manually onto SC-Leu and SC-Leu-Ura agar media. Plates were incubated for 24 hr and 48 hr at 30° and imaged. The growth of nearly all Ura^–^ clones was rescued in the *pre1-1 pre2-1* strain (Figure 1, and data not shown).

### Systematic growth analysis for degradation dependencies and quantification of growth

To simplify analyses and condense the library to a single 96-well plate, a subset of 77 unique plasmids that conferred *pre1,2*-dependent growth on SC-Leu-Ura was selected and subjected to further quantitative growth analysis (Figure S1). These 77 plasmids, along with plasmids expressing *URA3-HA-CL1* (pSM2288), URA3-HA-CL1* (pSM2337; CL1* is a CL1 variant with nine additional C-terminal residues (Metzger 2009), the positive control plasmid (pSM2697), which expresses *URA3-HA*, and the empty vector (pSM173) (six replicates of each control), were rearrayed in a randomized order into a single 96-well plate. Control plasmids were placed at various positions to control for differential conditions within a single plate; (Figure S1, A–D). Plasmids were transformed in 96-well format into WT and mutant strains, and selected on SC-Leu plates. Transformants (in quadruplicate) were replica pinned from source plates to solid SC-Leu and SC-Leu-Ura media using a manual pin tool, and incubated for 24 hr at 30°. The relative growth between each mutant and the WT was calculated as the Log_2_ ratio of the mean normalized colony size of mutant to WT for each degron. Relative growth is reported in the heatmaps for significant differences between mutant and WT, assessed by a Mann-Whitney test.

### Cycloheximide-chase and steady-state protein analysis

For cycloheximide chase analysis, saturated cultures were backdiluted to an OD_600_ of 0.3, and grown for 3–4 hr to mid-log phase. Cells were harvested and resuspended in fresh SC-medium at 2 OD_600_ units/ml. To initiate the chase, cycloheximide was added to 100 μg/ml, and, to terminate it, aliquots of cells were transferred to an equal volume of 20 mM sodium azide on ice at the indicated times. For steady-state protein analysis, samples were processed the same way, except that cycloheximide was omitted prior to collecting cells.

Preparation of protein lysates, SDS-PAGE, western blotting, antibodies used, and imaging are described in File S1.

### Proteasome inhibition with MG132 and ubiquitin E1 inactivation

Proteasome inhibition was performed based on the method of Liu *et al.* (2007), which optimizes the efficacy of MG132. Yeast were grown overnight in SCP– medium. Cultures were diluted to an OD_600_ of 0.5 in SCPS-medium and grown for 3–4 hr. Cells were harvested and resuspended in fresh SCPS-medium at 2 OD_600_ units/ml. Cultures were split and DMSO or MG132 (dissolved in DMSO) was added to a final concentration of 75 μM for 2 hr at 30° with shaking. Sodium azide was added to a final concentration of 10 mM, and cells were harvested. Protein extracts were prepared as described previously. To test the requirement of ubiquitylation on degron fusion protein levels, strains SM5923 (*UBA1*) and SM5925 (*uba1-204*) were grown at 25° and then shifted to 37° for 1 hr prior to addition of cycloheximide. Cells were incubated at 37° for an additional 45 min and processed for western blotting.

### Determination of protein half-lives and steady-state levels

Quantification of cycloheximide chase experiments was performed using QuantityOne software (BioRad). Signal from Ura3-HA-degron and the loading control (hexokinase) was quantified. The amount of Ura3-HA-degron at each time point was normalized to the amount of loading control protein at the same time point. The percent remaining was calculated from the normalized protein levels at each time point relative to the 0 min time point.

To determine the half-lives of proteins, we assumed that their degradation follows the first-order exponential decay model, *P* = 100e^(–λt)^, where *P* is the percent of protein remaining at time t, and λ is the decay constant. This model was fitted to the data by nonlinear least-squares regression analysis to obtain an estimate of λ. The half-life of the protein is then calculated from the estimate of λ. For comparisons of fitted curves, an *F*-test was used to generate a *P*-value. The *P*-values reported in plots have been adjusted to correct for multiple comparisons (Hochberg and Benjamini 1990) for all comparisons of protein decay from cycloheximide chase analyses reported in this study. Steady-state levels of Ura3-HA-degron proteins were quantified using Image J (NIH).

### Data availability

Strains and plasmids are available upon request. Table S3 contains peptide sequences of all degrons isolated in this study, with nucleotide sequences available upon request.

## Results

### A genetic screen to isolate degradation signals (degrons) that destabilize Ura3

To identify protein sequences that destabilize Ura3-HA in a proteasome-dependent manner, we generated a degron plasmid library by cloning DNA fragments into pSM2697 (Figure 1A and *Materials and Methods*) and transformed into yeast, selecting Leu^+^ transformants, followed by replica plating to medium lacking uracil. Our initial screening yielded 153 transformants (∼5% of total tested) that grew poorly, or not at all, after 24 or 48 hr on –Leu –Ura medium, indicative of either a destabilized Ura3-HA fusion protein, decreased expression, or lack of Ura3 function (Figure 1, B–D and data not shown; see also spot test growth of several examples, Figure 1F).

Because we were interested solely in plasmids expressing Ura3-HA-degron proteins whose stabilization is proteasome-dependent, clones were transformed into the proteasome mutant *pre1-1 pre2-1*, and tested for growth on medium lacking uracil. As expected, wild-type (WT) cells expressing the control degron construct Ura3-HA-CL1 failed to grow on SC-Leu-Ura plates, but growth was restored in the *pre1pre2* mutant (orange box, compare Figure 1, E–D; see also Figure 1G). We found that nearly all of the library clones were capable of growing on medium lacking uracil in the *pre1pre2* mutant (Figure 1, E *vs.* D and data not shown; see also spot test growth of several examples in Figure 1G), indicating that they express Ura3-HA-degron fusion proteins targeted for proteasomal degradation.

To examine degradation directly, we assessed whether the Ura3-HA fusion proteins in strains spotted in Figure 1, F and G were rapidly degraded by cycloheximide chase analysis (Figure 1H). Without a degron, Ura3-HA is stable over the time course of the chase experiment, with a projected half-life on the order of many hours, whereas the control construct Ura3-HA-CL1 is rapidly degraded with a half-life of 1.6 min, consistent with our previously published results (Metzger *et al.* 2008). Similarly, most clones isolated in our screen are also very rapidly degraded, many with half-lives of < 3 min (Figure 1H and data not shown). Because even a small amount of Ura3 might suffice for growth, and, since we screened for a Ura^–^ phenotype, we might not have isolated “slower-acting” or weaker degrons that promote half-lives of >5 min. For further analysis, we used a subset of 77 unique Ura3-HA-degron plasmid constructs (as determined by DNA sequencing), herein referred as the “degron library” (Figure S1 and Table S3). The degron peptides appended to Ura3-HA range from seven to 58 amino acids in length.

### Identification of Doa10-dependent and -independent degrons indicates several E3 ligases are involved in CytoQC of our degron constructs

**Figure 2:**
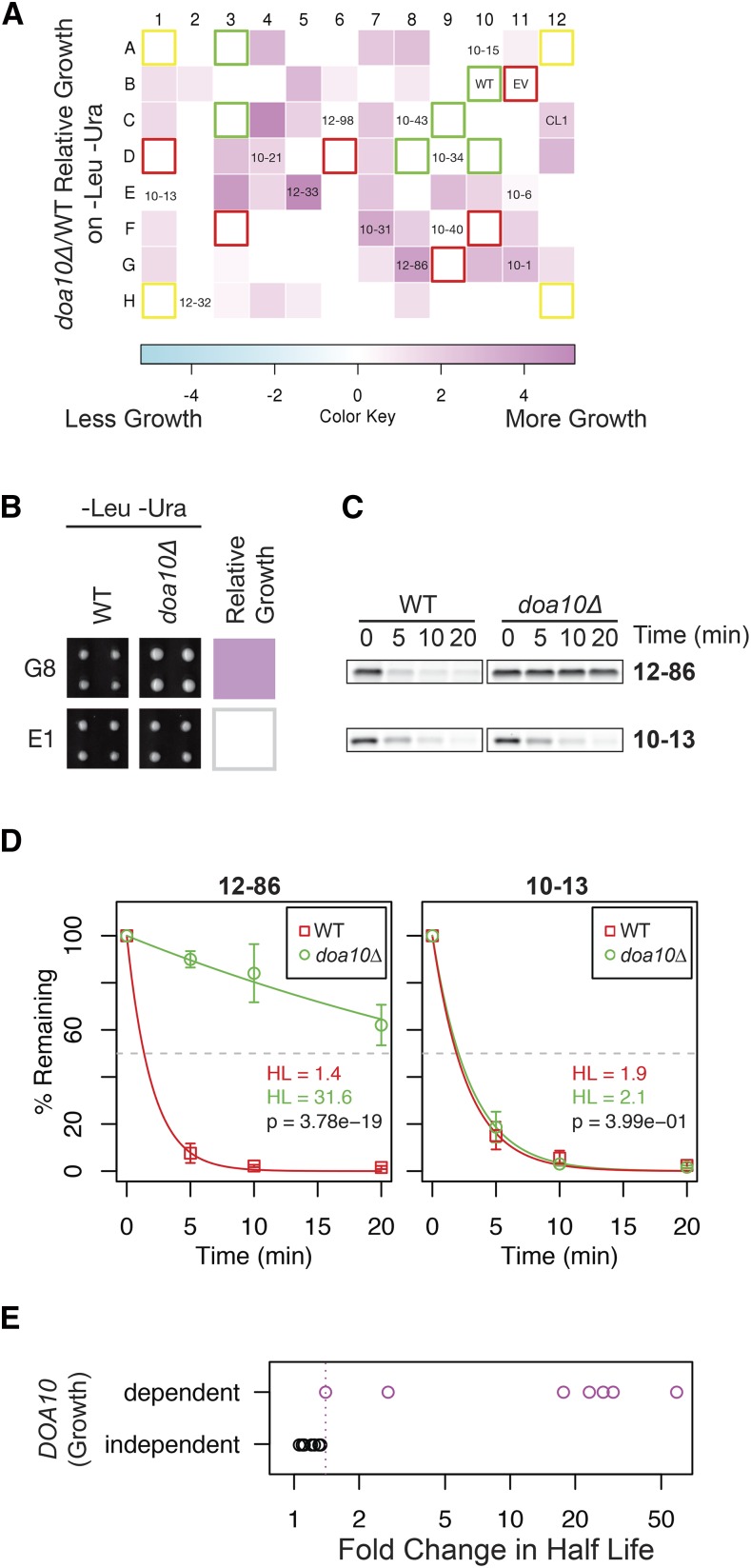
The degron collection contains both Doa10-dependent and Doa10-independent degrons. (A) Heatmap representation of the relative growth for *doa10*Δ and WT strains expressing the same degron. WT and *doa10*∆ strains (SM4460 and SM 4820) transformed with a subset of 77 clones, referred to here as the degron library, were rearrayed in a randomized order in 96-well format, pinned in quadruplicate from solid medium to solid medium, grown for 24 hr, and imaged (growth plates are shown in Figure S1 and also include the degron constructs containing CL1 and CL1*). The difference in growth between WT and *doa10*∆ strains was quantitated from the images and expressed as a heat map, in which the deeper the pink color the greater the growth differential between WT and *doa10*∆; white signifies no statistically significant difference in growth between WT and mutant. The positions of the 13 representative degron constructs comprising the “degron tester set” used in Figure 3 and later tests are indicated. Controls for growth express Ura3-HA (green boxes), no Ura3 (red boxes), or Ura3-HA-CL1 (CL1). Yeast in corner positions (yellow boxes) express Ura3-HA and were not used in the analysis. (B–D) Growth quantification and cycloheximide chase analysis for WT and *doa10*Δ expressing two sample degrons. The degrons examined are 12-86 from well G8, and 10-13 from well E1; these panels are taken from Figure S1 and enlarged. Chases were performed in triplicate, and the mean and 1 SD of the mean for each time point is shown. Half-lives and *P*-values were calculated as described in *Materials and Methods*. (E) The half-lives of the 13 tester set degrons and CL1 in *doa10*Δ and WT were determined by cyclohexamide chase (Figure S2), and their ratio was plotted along the *x*-axis of the strip chart. Degron constructs that show *DOA10*-dependent growth (pink circles), and *DOA10*-independent growth (black circles), are indicated. The pink dotted lines represents the smallest fold change in half-life between *doa10*Δ and WT that was detectable by growth for the 14 degrons analyzed.

We previously showed that Ura3-HA-CL1 is dependent upon Doa10 for degradation (Metzger *et al.* 2008), prompting us to ask how common Doa10-dependent degradation is among our 77-member degron library. Library and control plasmids were arrayed in 96-well format and transformed into WT and *doa10*∆ strains. To assess growth, transformants were replica-pinned in quadruplicate from this master plate onto –Leu and –Leu-Ura agar plates, and incubated for 24 hr at 30° (Figure S1, A–C). It should be noted that pinning from solid-to-solid medium gives more quantifiable detection of differential growth than the liquid-to-solid plating shown in Figure 1. Areas of the colonies were measured from digital images of the plates using image-analysis software (Carpenter *et al.* 2006; Lamprecht *et al.* 2007; Vokes and Carpenter 2008), and normalized within each plate to the control colonies expressing no Ura3 or Ura3-HA (Figure S1, A–C, red and green boxes, respectively). The relative difference in growth between WT and the *doa10*∆ mutant was calculated from the normalized areas and displayed as a heatmap, where pink signifies more growth in *doa10*∆ than WT, and white signifies no difference in growth between these two strains (Figure 2A; also repeated for ease of analysis as Figure S1D).

As expected for strains expressing Ura3-HA-CL1, growth of the *doa10*∆ transformant was significantly greater than the WT transformant (Figure 2A, position C12; Figure S1, B and C). Notably, similar to Ura3-HA-CL1, approximately half of the degron library transformants showed increased colony size in *doa10*Δ compared to WT, suggesting that the Doa10 E3 ligase is involved in the turnover of many Ura3-degron fusion proteins. Thus, Doa10 appears to have a widespread role in CytoQC, in addition to its well-characterized roles in ERAD and nuclear quality control. However, since many degrons showed no difference in growth in *doa10*∆ *vs.* WT (Figure 2A, white squares; see also Figure S1, A–C), other E3 ligases may be involved in the turnover of those degron-bearing constructs. These could function redundantly with Doa10, or may define a completely Doa10-independent pathway(s) for degradation of many of the Ura3-HA-degron constructs. It should be noted that Hrd1, the other ERAD E3, does not appear to act on any members of the degron library (data not shown).

To confirm that poor growth in our quantitative growth assay indeed reflects protein turnover, we examined degradation of a predicted Doa10-dependent (12-86; G8) and Doa10-independent (10-13; E1) Ura3-HA-degron protein. Consistent with their growth patterns, 12-86 was strongly stabilized in the *doa10*∆ strain by cycloheximide chase, whereas 10-13 was degraded as efficiently as in WT (Figure 2, B–D). Thus, the quantitative growth assay provides a reliable method to determine which gene products are involved in the turnover of constructs in our degron library. Quantitative growth of the entire degron collection in a variety of E3 mutant strains is summarized in Figure S1E.

### A “degron tester set” requires ubiquitylation for proteasome-mediated degradation

**Figure 3:**
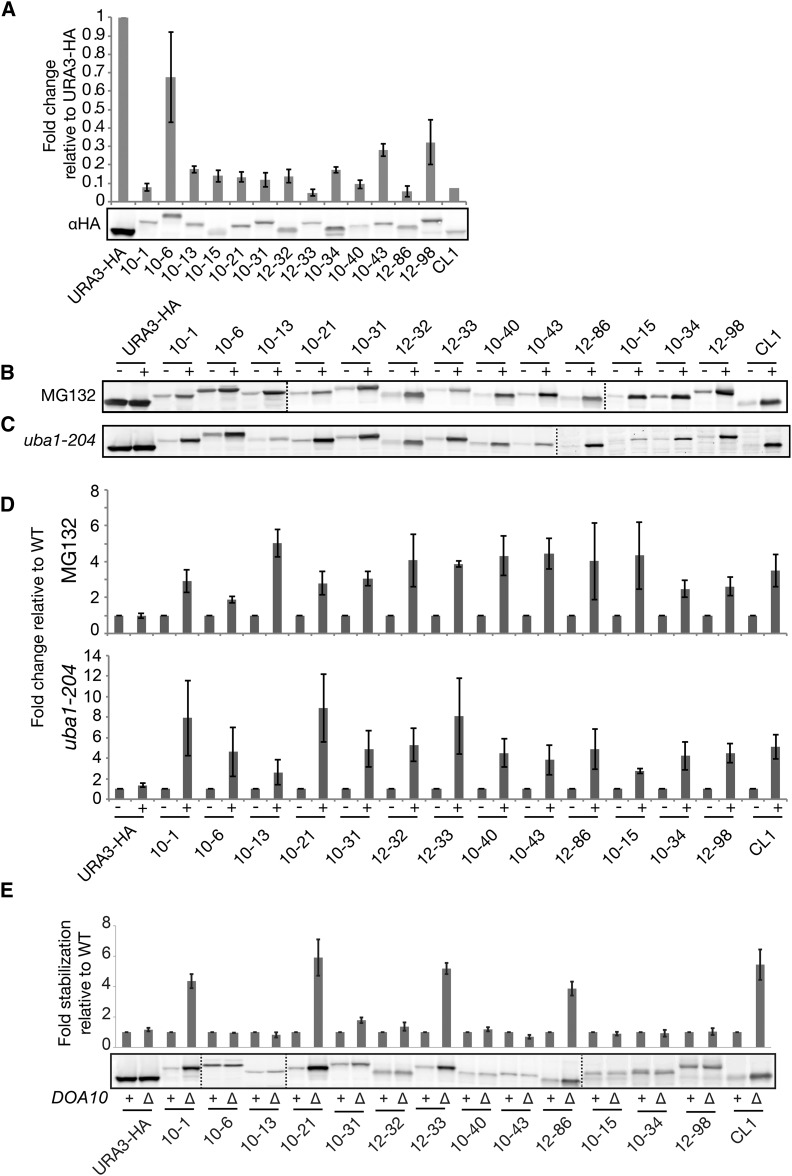
The “degron tester set” constructs all show ubiquitin- and proteasome-dependent degradation. A representative subset of Ura3-HA-degron proteins from the degron library was examined by western analysis. (A) Ura3-HA-degron fusion proteins isolated in the screen differ in migration rate according to their predicted lengths, and are present at lower levels than Ura3-HA, in WT cells (SM4460), reflecting degradation. Steady-state protein extracts were prepared in triplicate, analyzed by SDS-PAGE and western analysis with anti-HA antibodies, as described in *Materials and Methods*, and a representative gel is shown. Quantitation of the HA signal was performed using ImageJ software, and normalized to a hexokinase control (not shown). The amount of normalized signal for degron-containing Ura3-HA-degron proteins relative to Ura3-HA with no degron (set to 1) is plotted. (B–D) Degradation for all degron constructs exhibit ubiquitin and proteasome dependence. In (B), WT cells expressing Ura3-HA or Ura3-HA-degron fusion proteins were pretreated with drug vehicle (–) or MG132 (+) for 2 hr prior to sample preparation. Proteins were detected by western blotting and quantified as in (A). In (C), degron constructs were expressed in a WT *UBA1* strain (–) or a mutant *uba1-204* strain (+) that is defective in ubiquitylation at nonpermissive temperature (Ghaboosi and Deshaies 2007). Cells were processed as described in *Materials and Methods*. (E) Doa10-dependent steady-state levels were measured in the WT strain SM4460 (+) and *doa10*∆ mutant SM4820 (–). Error bars represent 1 SD of the mean. Vertical dotted lines indicate where separate gels are spliced together.

For the remainder of this study, we chose a representative set of 13 degron constructs for in-depth analysis based on different degron lengths, hydrophobicities (Table S3), and growth rates in WT and E3 ligase mutants (Figure 1D and Figure S1E). We refer to these as the “degron tester set” (Figure 3A and Table 1) and we analyzed them together with the previously characterized degron CL1. As predicted by their growth patterns, the steady-state level of all the Ura3-HA-degron proteins was significantly lower in WT cells, as compared to Ura3-HA with no degron. In addition, the observed migration by SDS-PAGE correlated with the predicted increase in molecular weight due to the presence of degron peptides of varying lengths (Figure 3A). Five members of the degron tester set, along with CL1 were predicted by growth to be Doa10-dependent (10-1, 10-21, 10-31, 12-33, and 12-86) and the remaining eight to be Doa10-independent (Figure 2, A and E). This Doa10-dependent or -independent degradation for all 13 members of the tester set was confirmed by cyclohexamide chase (Figure S2) and steady-state analysis (Figure 3E).

**Table 1:** Degron tester set sequences and properties

Degron	Sequence	Length (# of aa)	Hydrophobicity (GRAVY)
CL1	ACKNWFSSLSHFVIHL	16	0.569
10-1	KSVTLESRSPKFLNWFSVFSLFKVITTG	28	0.268
10-6	DFFFLFVLPSEQKVKSPECDKDILRLTITQVLSHKTPYI	39	0.010
10-13	CSEIIPMSRSTPISTMG	17	0.212
10-15	SYWLTVY	7	0.429
10-21	ENQEGLLKFQSIFVYCYRLLLKTLPL	26	0.288
10-31	SIFYHIGTDLWTLSEHYYEGVLSLVASVIISGR	33	0.533
10-34	VVLVVVF	7	3.943
10-40	YMSILRCASGKISIAAPPYIF	21	0.829
10-43	QSHMTIESKTRIERKMLVCTPG	22	−0.586
12-32	LWLEDLQRTVVLIMVKPG	18	0.650
12-33	AGESFNFMVKLLYKHPILPCLKTLLSIRSSCSPR	34	0.241
12-86	VSFAFNLNSLIVGILRFHW	19	1.126
12-98	SQAFIATLLFDSSMSALPIIPKQNSVSVGLFTH	33	0.664

Proteasome-mediated degradation generally requires ubiquitylation of the substrate; however, ubiquitin-independent signals also exist (Erales and Coffino 2014). To determine whether degradation of our degron-containing proteins was ubiquitin-dependent, we analyzed levels of the Ura3-HA-degron constructs in the *uba1-204* mutant, which encodes a temperature-sensitive mutation of the sole E1 ubiquitin activating enzyme (Ghaboosi and Deshaies 2007), and is required for ubiquitin conjugation. At the nonpermissive temperature, the Ura3-HA-degron protein levels were around two- to eight-fold higher in the *uba1-204* strain than in the WT strain at the nonpermissive temperature (Figure 3, C and D, bottom). This trend of stabilization was mirrored in cells treated with the proteasome inhibitor MG132 (Figure 3, C and D, top). Together, these data indicate that degradation of all members of the degron tester set require the full ubiquitin-proteasome pathway.

### All members of the degron-tester set require Hsp40 (Ydj1) and Hsp70 (Ssa1/2) for degradation

**Figure 4:**
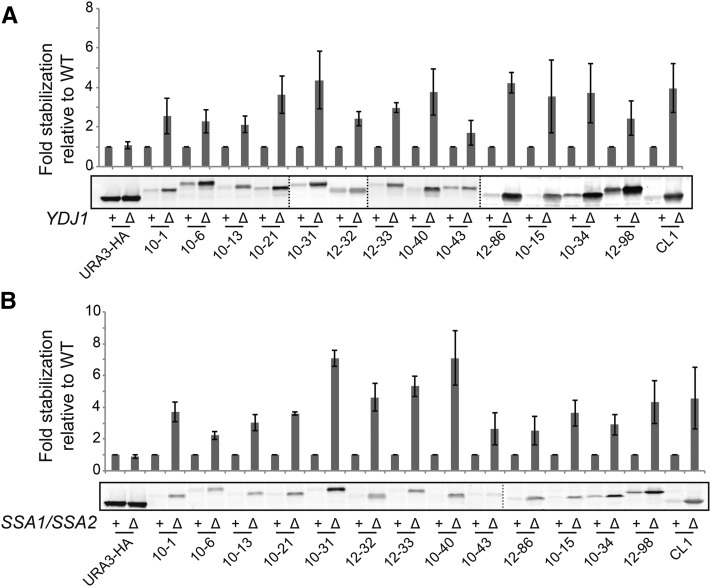
The cochaperone and chaperone proteins Hsp40 and Hsp70 are required for efficient degradation of Ura3-degron proteins. (A, B) Steady-state western blots of Ura3-HA and Ura3-HA-degron proteins in WT (+) or deletion mutants (Δ) for *ydj1*Δ (SM 4460 and SM4819) (A) and *ssa1*Δ *ssa2*Δ (SM5755 and SM5774) (B). Quantification of Ura3-HA and Ura3-HA-degron proteins in the deletion mutants relative to WT is shown. Error bars represent 1 SD of the mean. Vertical dotted lines indicate where separate gels are spliced together.

The chaperone Hsp70 and its cochaperone Hsp40 have been shown to play a critical role in the degradation of numerous ERAD-C and CytoQC substrates (Heck *et al.* 2010; Metzger *et al.* 2008; Nakatsukasa *et al.* 2008; Prasad *et al.* 2010; Shiber *et al.* 2013). These chaperones may act upstream or downstream of E3 ubiquitin ligase activity (or both), but prior to degradation by the proteasome. Interestingly, deletion of *SSA1/SSA2* and *YDJ1* stabilized every degron-containing protein in our tester set, albeit to varying extents (Figure 4). The observation that every degron construct requires the Hsp70s and Hsp40 for turnover, whereas their E3 ligase dependency for degradation varies, suggests that the discrimination step for these distinct CytoQC substrates is mediated by E3 ligases rather than the Hsp40 or Hsp70 chaperones.

### The E3 ligases San1 and Ubr1 only very modestly affect the degradation of a small subset of degron-containing proteins

**Figure 5:**
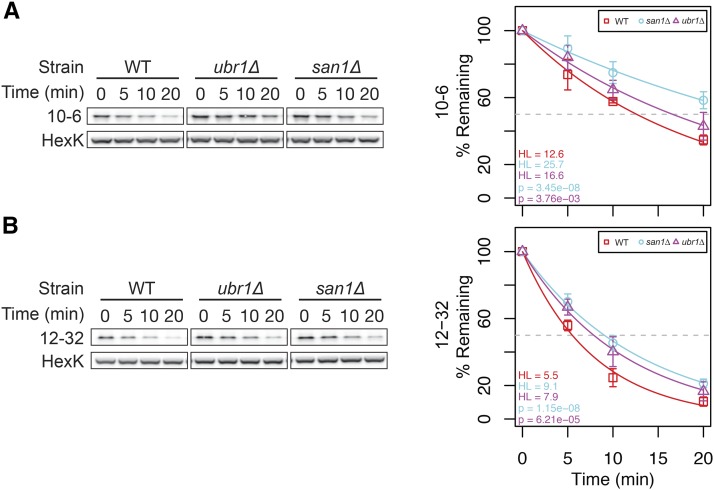
Lack of *UBR1* or *SAN1* has only a minor effect on the degradation of a few Ura3-HA-degron proteins in the tester set. (A) and (B). Cyclohexamide chase and quantitation are shown for degron constructs 10-6 and 12-32, respectively, which showed slightly improved growth in the single mutants *ubr1*∆ (SM5745) and *san1*∆ (SM5746) *vs.* WT (SM4460). Hexokinase (HexK) is the loading control.

In addition to Doa10, other E3 ligases have been implicated in CytoQC degradation. Mutations in *SAN1* and/or *UBR1* have been reported to stabilize a variety of CytoQC substrates (Eisele and Wolf 2008; Guerriero *et al.* 2013; Heck *et al.* 2010; Nillegoda *et al.* 2010; Park *et al.* 2013; Prasad *et al.* 2010, 2012; Summers *et al.* 2013; Khosrow-Khavar *et al.* 2012). Since many of our degron-containing proteins are degraded efficiently in *doa10*∆ cells, we wanted to determine if San1 and Ubr1 are involved in their turnover. A small number of constructs in the degron library showed a minor increase in growth in the *san1*∆ and *ubr1*∆ mutants (Figure S1E). We further examined by cycloheximide chase the stability of two of these that are members of the degron tester set, 10-6 and 12-32 (Figure 5). Loss of Ubr1 or San1 only very slightly increased the half-life of 10-6 and 12-32, as compared to the WT strain (for 10-6 half-life increased from 12.6 min in the WT strain to 16.6 and 25.7 min in the Ubr1 and San1 mutants, respectively, and for 12-32, half-life went from 5.5 min in the WT strain to 7.9 and 9.1 min in the mutants), consistent with the modest differences observed in the growth test. Furthermore, steady-state analysis of these and other members of the tester set in the double mutant *san1*∆ *ubr1*∆ revealed no additional stabilization (Figure S3). We conclude that the San1 and Ubr1 E3 ligases do not play a major role in the ubiquitylation of our degron constructs. This finding is in contrast to *doa10*∆, which almost completely stabilizes some of the degron constructs (cf. Figure 5 to Figure S2, and Figure S3 to Figure 3E).

### The ubiquitin E3 ligase Ltn1 affects the degradation of some members of the degron tester set

**Figure 6:**
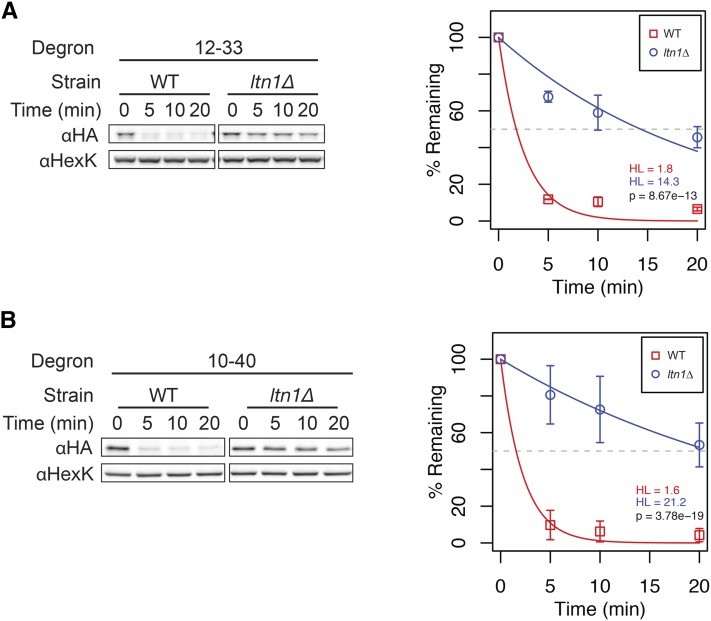
Lack of *LTN1* affects the degradation of several Ura3-HA-degron proteins in the tester set. Western blots and quantitation of cycloheximide chase analysis of WT (SM4460) and *ltn1*Δ (SM5559) strains expressing the Ltn1-dependent degrons 12-33 and 10-40 [(A) and (B), respectively].

Ltn1 (also called Rkr1) is an E3 ligase shown to be part of the ribosomal quality control (RQC) complex. It is required for the ubiquitylation and degradation of nonstop polypeptides and proteins containing translation pause-inducing sequences, such as polylysine or polyarginine stretches (Bengtson and Joazeiro 2010; Brandman *et al.* 2012; Shen *et al.* 2015; Wilson *et al.* 2007). In a separate study, we identified *LTN1* in a screen for deletion mutants that stabilize a degron construct related to Ura3-HA-CL1, called CL1*, (Metzger 2009), which is not a nonstop protein and does not contain a polybasic stretch. Given its importance in Ura3-HA-CL1* PQC, we asked whether the *ltn1*∆ mutant also would stabilize proteins in our degron library. Comparison of the steady-state level of degron constructs in WT *vs.* *ltn1*∆ suggest that Ltn1 contributes to the turnover of many of the degron constructs (10-13, 10-31, 10-15, 10-34, 10-40, 12-33, and CL1; Figure S3B), as does the quantitative growth test (Figure S1E). Two of these constructs, 10-40 and 12-33, were further examined by cycloheximide chase analysis. As expected, both, showed significant stabilization in *ltn1*∆, as compared to WT (Figure 6). In contrast, degron construct 10-6, which showed only very slight stabilization in *ltn1*∆ by growth and steady state analysis (Figure S1E and Figure S3B) also showed only slight stabilization in ltn1∆ by cycloheximide chase (data not shown). It should be noted that, for some constructs for which improved growth was seen in *ltn1*∆, in particular 10-43, we observed no difference in stability of the degron construct between WT and *ltn1*∆, although a high molecular weight (HMW) species was apparent in the *ltn1*∆ mutant (Figure S3B and Figure S4; see *Discussion*).

### Ltn1 appears to affect Ura3-HA-degron constructs by a mechanism distinct from translational pausing

Recently, several groups have provided insight into the mechanism by which the RQC operates. Ltn1 associates with ribosomes that are translationally stalled due to the presence of polylysine/arginine pause signals in the nascent polypeptide, or due to the synthesis of polylysine from the poly(A) tail of an mRNA lacking a stop codon. In both cases, Ltn1 is well-positioned to ubiquitylate the emerging translation product (Bengtson and Joazeiro 2010; Brandman *et al.* 2012; Lyumkis *et al.* 2013; Lyumkis *et al.* 2014; Shao *et al.* 2015; Shen *et al.* 2015). However, our degron constructs differ from the published Ltn1-dependent substrates, by having intact stop codons, and not encoding polylysine or other polymeric repeats (Table S3). Although it remains possible that some degron sequences form stem-loop structures with the 3′ UTR, leading to translational stalling, it seems unlikely given that numerous degrons having diverse sequences are affected in the *ltn1*∆ mutant.

**Figure 7:**
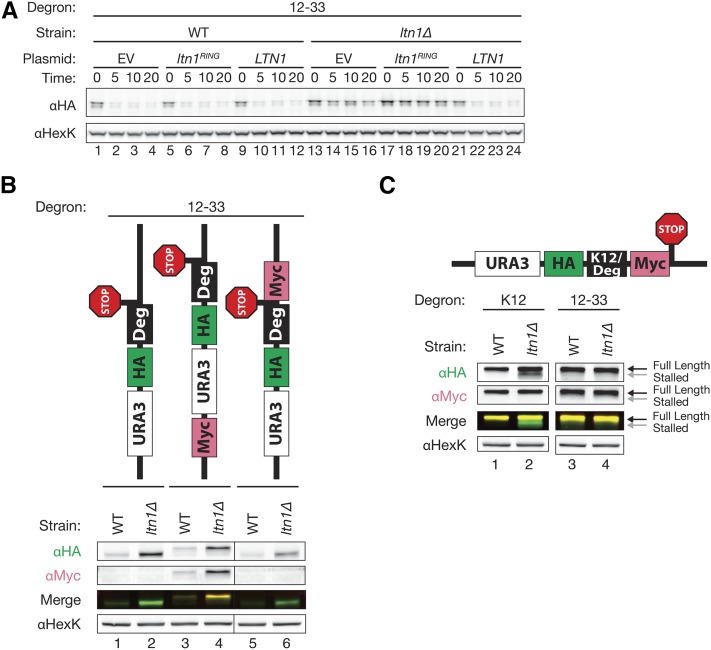
Ltn1-mediated degradation of the Ura3-HA-degron protein 12-33 requires E3 ligase activity, and does not involve production of a nonstop protein or a translational pause signal (A) The ubiquitin ligase activity of Ltn1 is necessary for the degradation of an *LTN1*-dependent degron. Cycloheximide chase analysis of the Ura3-HA-12-33 degron construct expressed from plasmid pSM2743 in an *ltn1*Δ strain (SM5559) bearing plasmid-borne *LTN1* (pSM2658), no *LTN1* (EV; pSM171), or the Ltn1 RING domain mutation C1508A (*ltn1^RING^*; pSM2659). (B) Translation of Ura3-HA-12-33 correctly terminates at the intended stop codon. HA and Myc epitopes were individually probed in steady-state western blots of the original construct (lanes 1-2), an N-terminally Myc-tagged derivative (lanes 3–4), or a derivative with Myc placed C-terminal to the stop codon, all expressed in WT (SM4460) or *ltn1*Δ (SM5559). In the merged image, HA and Myc signals are displayed in the green and red channels, respectively; protein containing both the HA and Myc epitopes appears yellow. (C) A stalled, truncated form of the Ura3-HA-Deg-Myc construct (as indicated by the presence of HA staining, but a lack of Myc staining) accumulates in *ltn1*Δ when the degron sequence is replaced with the known stall-inducing sequence (K12), but not with degron 12-33 (lanes 2 *vs.* 4, respectively). The Myc coding sequence shown in the schematic is immediately downstream of, and in frame with, K12 or degron 12-33.

To investigate the basis for Ltn1-dependent degradation of our constructs, we carried out further experiments with degron 12-33. We first examined whether the ubiquitin ligase activity of Ltn1 was required for degradation. Ura3-HA-12-33 was degraded in the WT strain (Figure 7A, lanes 1–12) and in the *ltn1*∆ mutant expressing plasmid-borne WT *LTN1* (Figure 7A, lanes 21–24), but stabilized in *ltn1*∆ cells with an empty vector or expressing *ltn1^C1508A^*, a RING mutant devoid of E3 ubiquitin ligase activity (Figure 7A, lanes 13–20) (Bengtson and Joazeiro 2010; Braun *et al.* 2007). We found a similar result for degron 10–40 (data not shown). Thus, the ubiquitin ligase activity of Ltn1 is critical for the degradation of our constructs.

Next, we wished to ask whether the stop codon in Ura3-HA-12-33 is indeed functional, rather than resulting in a nonstop protein. To do so we placed a Myc epitope immediately downstream of the stop codon, or at the N-terminus as a control. As expected, the parental construct Ura3-HA-12-33 without a Myc tag was detected with anti-HA antibodies (and not with anti-Myc), and showed increased steady-state levels in *ltn1*∆ *vs.* WT (Figure 7B, lanes 1 and 2). The addition of Myc at the N-terminus of Ura3-HA-12-33 resulted in a protein with a noticeable size shift (Figure 7B, lanes 3 and 4) that is stabilized in *ltn1*∆, and is detectable with both anti-HA and anti-Myc antibodies (Figure 7B, αHA, αMyc, and merge panels, yellow band). Importantly, when Myc was placed immediately downstream of the stop codon in Ura3-HA-12-33, no size-shifted, Myc-tagged product is evident; and indeed the stabilization pattern is indistinguishable from that of the parental construct (Figure 7B, cf. lanes 5 and 6 to lanes 1 and 2). Similar results were also observed for Ura3-HA-10-40 (data not shown). Taken together, these findings indicate that translation of Ura3-HA-12-33 (and Ura3-HA-10-40), correctly terminates at the intended stop codon, and that its recognition by Ltn1 must therefore not involve nonstop polypeptide production.

Finally, we wished to examine whether degron 12-33 promotes ribosome stalling, which can result in truncated translation products that are rapidly degraded in WT cells, but are stable when *LTN1* is deleted (Bengtson and Joazeiro 2010; Brandman *et al.* 2012; Shen *et al.* 2015). We placed an in-frame Myc epitope just C-terminal to degron 12-33. As a control, we replaced the 12-33 degron with a stretch of 12 lysine codons (K12) (Figure 7C), known to induce translational arrest and Ltn1-dependent protein degradation (Bengtson and Joazeiro 2010; Braun *et al.* 2007). These constructs allowed us to ask whether we could detect only fully translated proteins containing both the HA and Myc epitopes, or whether we could also detect truncated proteins containing solely HA (and not Myc), indicative of a ribosome stalling sequence upstream of Myc. Consistent with previous results (Bengtson and Joazeiro 2010; Brandman *et al.* 2012; Shen *et al.* 2015) expression of *URA3-HA-K12-Myc* gave rise to two species of proteins: a slower migrating species detectable with both anti-HA and anti-Myc antibodies, and a faster migrating form detectable with anti-HA but not anti-Myc antibodies (Figure 7C, left panels, lane 2). This faster migrating species (Figure 7C, labeled “stalled”) results from arrested translation due to the polylysine tract, and is observed only when it is stabilized in *ltn1*∆ (Figure 7C, lanes 1 and 2). The slower-migrating protein species detected with both anti-HA and anti-Myc antibodies represents the full-length protein that is produced from translation through the pause site. By contrast, we failed to observe any truncated species in either WT or *ltn1*∆ for our degron construct 12-33 (Figure 7C, lanes 3 and 4) or 12-40 (data not shown), indicating that translation of these degron sequences apparently do not cause the ribosome to stall.

Interestingly, there were equivalent levels of the full-length Ura3-HA-Deg12-33-Myc in the WT and the *ltn1*∆ strains (Figure 7C, lanes 3 and 4). Thus, the 12-33 sequence does not function as a degron when it is not the most C-terminal element of the protein. Similar results were seen for degron 10-40 (data not shown). Taken together, our results demonstrate that Ltn1 can mediate the degradation of proteins other than nonstop proteins and ribosome pause-inducing proteins, and suggest that Ltn1 activity may require that the degron be at the very C-terminus of the protein.

## Discussion

A major focus in the proteostasis field is to define the recognition codes that direct particular substrates to specific E3 ligases (Deshaies and Joazeiro 2009; Fredrickson and Gardner 2012; Metzger *et al.* 2008; Ravid *et al.* 2006). Studying PQC using naturally occurring misfolded proteins is complicated by the fact that the degradation signal is unknown, could exist anywhere in the protein, and multiple degradation signals are likely to be present in a single unfolded protein. Degrons are signals, often in the form of a peptide, defined by their ability promote a protein’s degradation (Varshavsky 1991). In previous studies, a library of peptides appended to a nuclear-directed form of GFP revealed fundamental principles of nuclear PQC (Fredrickson *et al.* 2011, 2013). Here, to facilitate the analysis of CytoQC and to build on an earlier screen by Gilon and coworkers (Gilon *et al.* 1998, 2000), we generated a comprehensive collection of peptides that possess degron function when appended to Ura3. Having many different short sequences fused to the same cytosolic reporter protein will simplify the analysis of CytoQC recognition signals, by limiting variation to a small, defined region of the substrate.

For creating this CytoQC degron library, we chose HA-tagged Ura3 as the reporter protein because it is cytosolic and genetically tractable. Sheared DNA fragments were cloned into the C-terminus of Ura3-HA. Degrons were identified as sequences that confer a defective growth phenotype on –Ura medium in a WT strain, but for which growth is spared in a proteasome mutant. This rescued growth when the proteasome is impaired indicates that Ura3 is functional, and thus unlikely misfolded, and that the degradation signal lies within the cloned degron sequence. Our degron collection contains 77 Ura3-HA-degron constructs ranging in size from seven to 58 amino acids in length (Table S3). For detailed analyses we focused on a representative “tester set” of 13 degron constructs. For most of these, the half-life is extremely short (<3 min). This degradation rate is considerably faster than that for previously reported model misfolded CytoQC substrates, such as Ura3-2 and Ura3-3, ∆2GFP, ∆ssPrA, ∆ssCPY*GFP, slGFP, NBD Ste6*, and a variety of Ts^–^ mutant proteins, which have half-lives ranging from 10 to 30 min (Eisele and Wolf 2008; Guerriero *et al.* 2013; Heck *et al.* 2010; Nillegoda *et al.* 2010; Park *et al.* 2013; Prasad *et al.* 2010, 2012; Summers *et al.* 2013; Khosrow-Khavar *et al.* 2012). The consistently rapid degradation we observe for members of our Ura3-HA-degron library may reflect a bias imposed by our screening criteria for a Ura– phenotype, which could have demanded an exceedingly low level of protein, a situation that may only be achieved for constructs that are very rapidly degraded. Alternatively, the positioning of degrons at the C-terminus of well-folded Ura3 may allow ideal exposure to E3 ligases, hastening their ubiquitylation and degradation. Because members of our degron collection are degraded so soon after synthesis, they may prove advantageous for identifying components that act on nascent proteins just after their release from the ribosome, rather than machinery that acts on other types of CytoQC substrates, such as previously well-folded proteins that have sustained damage or become unfolded. It will be of interest to test positional effects of our degrons and their transferability to other reporter proteins. To date, we have examined degrons CL1, 10-1, 10-13, and 10-43, and all retain their degron properties when transferred to the C-terminus of GFP (data not shown).

To determine the E3 recognition pattern for all members of our degron library, we tested for restored growth on –Ura medium in the E3 mutants for the E3s most prominently implicated in CytoQC to date, which include Doa10, Ubr1, San1, and Ltn1 (Figure S1E). For members of the tester set, we also directly determined their half-lives in WT and mutant cells, and their steady-state levels (Figure 2, Figure 3, Figure 4, Figure 5, Figure 6, Figure S2, and Figure S3). It is notable that of all the E3 mutants tested, the *doa10*∆ mutant shows the most profound effect on stabilization, and acts on about half of our degron library constructs. The *ltn1*∆ mutant also stabilizes numerous constructs, but stabilizes them to a lesser extent than *doa10*∆. Notably, only modest effects were seen for *ubr1*∆ (or *ubr2*∆) and *san1*∆, with the growth of far fewer degron constructs affected in these latter mutants, and to a lesser extent. Below, we discuss in depth our findings with these E3s. We note that we have not yet tested the E3s Hul5 and Rsp5, which were shown to act on cytosolic proteins that are destabilized by heat shock (Fang *et al.* 2011, 2014). Nor have we examined the E3 ligase Hel2, shown to play a broad role in nascent protein CytoQC (Duttler *et al.* 2013). However, it will be of interest to study mutants defective for these and many other candidate UPS components.

Doa10, which localizes to the ER membrane and the INM, plays a key role in ERAD-C in which it ubiquitylates membrane proteins with a cytosolic lesion, and it was recently shown to mediate prERAD—a process in which the cytosolic precursors of some post-translationally secreted proteins that fail to be translocated are degraded (Ast *et al.* 2014). In addition, Doa10 acts on the nuclear proteins MATα2 and misfolded Ndc10 (Deng and Hochstrasser 2006; Furth *et al.* 2011; Ravid *et al.* 2006). Previous findings by us and others showing that Doa10 also mediates the ubiquitylation of the CytoQC substrate Ura3-HA-CL1 raised the intriguing possibility that the ER or nuclear membrane may serve as a major platform for CytoQC (Gilon *et al.* 1998; Metzger *et al.* 2008; Ravid *et al.* 2006). Here, we found here that many (about half), of our degron-containing proteins show improved growth in *doa10*∆ (Figure 2, Figure 3E, and Figure S1), suggesting that many CytoQC substrates in addition to Ura3-HA-CL1 may also be ubiquitylated at the face of the ER membrane or possibly the INM. Thus the ER/INM may serve as a convenient way to organize the cellular machinery used for ubiquitylation of a substantial number of substrates, although clearly not all, since importantly, many of our degron constructs show no stabilization in *doa10*∆ mutant (Figure 2, Figure 3E, Figure S1, and Figure S2). The mechanism whereby CytoQC substrates are delivered to the membrane for degradation presents an interesting problem, and could involve specific guide or escort proteins that our degron constructs could help to identify. Alternatively, Doa10-dependence for the degradation of certain constructs might reflect the possibility that these constructs are synthesized on ER-bound ribosomes (Kraut-Cohen *et al.* 2013; Reid and Nicchitta 2015), and, thus, Doa10 would be among the first E3s that they encounter.

Another E3 ligase that affected the stability of many members of the degron library was Ltn1, recently shown to be a key component of the RQC complex (composed of Ltn1, Rqc1, Rqc2/Tae2) (Brandman *et al.* 2012; Shen *et al.* 2015). Ltn1 associates with the 60S ribosomal subunit, and ubiquitylates incompletely synthesized nascent proteins that are translationally stalled due a faulty mRNA, or the presence of a ribosome pause sequence such as a polybasic tract of amino acids (Brandman *et al.* 2012). Several of the degron constructs in our tester set are stabilized in the *ltn1*∆ mutant, as evidenced by steady-state or cycloheximide chase analysis (Figure 6 and Figure S3). We examined degron 12-33 in depth, and showed that this sequence does not cause ribosome pausing, in contrast to what we see for a classical engineered polylysine pause sequence (K12) (Figure 7D). Furthermore, the gel mobility of the Ua3-HA-12-33 degron fusion protein is considerably greater than that of Ura3-HA (Figure S3B), suggesting that the entire protein is synthesized (including the degron sequence), and therefore is likely to be completely released from the ribosome. Thus, Ltn1 may have a PQC role for nascent proteins that have been newly released from the ribosome, or may influence certain aspects of cellular proteostasis, in addition to its well-characterized role in handling paused proteins that remain associated with the 60S ribosomal subunit.

It is notable that, for one of our tester set degron constructs, Ura3-HA-10-43, we saw significantly improved growth in *ltn1*∆ *vs.* WT, despite observing only minor stabilization of the degron construct. Instead, we observed a HMW protein species that is present in the *ltn1*∆ mutant, but is absent in WT cells (Figure S4, A–C; note that the HMW species is sometimes observed as a discrete band or as multiple bands). The HMW bands are also absent when the other RQC components, Rqc1 and Rqc2/Tae2, are absent (Figure S4D). The additional forms of Ura3-HA-10-43 likely account for the improved growth on –Ura medium. We hypothesize this HMW species may correspond to carboxy-terminal Ala and Thr extensions (‘CAT” tails), that have been shown to arise from nontemplated addition of these amino acids, and is mediated by the Rqc2 component of the RQC complex (Shen *et al.* 2015). Thus, 10-43 and other degrons in our library that show HMW species in the *ltn1*∆ mutant, may provide insight into signals that prompt this unusual modification. Overall, it is important to understand the nature and breadth of Ltn1 substrates, as a mutation in the mouse *LTN1* homolog, *lister*, results in a neurodegenerative disorder similar to spinal-bulbar muscular atrophy (SBMA) and amyotrophic lateral sclerosis (ALS) (Chu *et al.* 2009). A more thorough understanding *LTN1* function could provide insight into human neurodegenerative disorders.

Two other E3s that have been implicated in CytoQC are Ubr1 and San1. For some well-studied model substrates, full stabilization is observed only in mutants deleted for both, while some CytoQC substrates appear to be ubiquitylated mainly by one or the other of these E3s (Eisele and Wolf 2008; Guerriero *et al.* 2013; Heck *et al.* 2010; Nillegoda *et al.* 2010; Park *et al.* 2013; Prasad *et al.* 2010, 2012; Summers *et al.* 2013). While Ubr1 is cytosolic, San1 is nuclear (Fredrickson and Gardner 2012). The San1-dependent degradation observed for many CytoQC substrates, along with the direct observation of nuclear localization of these substrates, has provided evidence that nuclear import contributes to their ubiquitylation and degradation. Interestingly, very few members of our degron library showed any dependence on either of these E3s for degradation, and the two that did (10-6 and 12-32) showed only very modest stabilization in either the *ubr*∆*1* or *san1*∆ single or the double mutants (Figure 5, and also Figure S1E and Figure S3). It is worthwhile pointing out that most San1 and Ubr1 substrates characterized to date have fairly long half-lives, whereas all of our degron constructs are exceedingly short-lived, as discussed above. It may be that our degron constructs are degraded so soon after being released from the ribosome that they may never have the chance to encounter Ubr1 or be transported to the nucleus, where San1-dependent degradation occurs.

In addition to E3s, chaperones play a key role in PQC, either by attempting to fold misfolded proteins, or by preventing misfolded proteins from aggregating so that they remain accessible to the PQC machinery (Chen *et al.* 2011; McClellan *et al.* 2005a, 2005b; Metzger *et al.* 2008). The PQC substrates, Ura3-CL1 and Ste6* are not ubiquitylated in the absence of the Hsp40 Ydj1 or the Hsp70 Ssa1p, indicating that this cochaperone/chaperone pair functions upstream of the ubiquitylation machinery (Metzger *et al.* 2008; Nakatsukasa *et al.* 2008), in some way assisting in delivery to the E3. We observed dependency on Ydj1 and Ssa1 for degradation for all of the degron-containing proteins in our tester set (Figure 4). This finding suggests that Ydj1 and Ssa1 may not provide specificity for directing substrates to a particular E3, but it clearly indicates that these chaperones are very important for a variety of CytoQC substrates. It will be interesting to determine if the role of Ydj1 and Ssa1 for our Ura3-degron constructs is to maintain their solubility so that they can be delivered to, or recognized by, a particular subset of E3s.

Most of the sequences we isolated as degrons originate from ORFs, but are out-of-frame translations (including reverse complement), or are from nonprotein-coding sequences such as rRNA genes and intergenic sequences. Thus, the majority of the peptides we isolated as degrons are of sequences that do not naturally exist in the yeast proteome, as is also the case for the degron CL1 (Metzger *et al.* 2008). Nevertheless, our degrons are still expected to be informative, since degron recognition within aberrant proteins is unlikely to be at the level of primary sequence, but instead probably involves physical characteristics such as exposed hydrophobicity or propensity to aggregate. It is notable that at least one human disease results from a frame shift mutation that creates a novel degron (Su *et al.* 2011). Other collections of short peptides with degron function exist, also constructed by fusing synthetic sequences or genomic DNA or cDNA to reporter proteins (Fredrickson *et al.* 2011; Gilon *et al.* 1998; Sadis *et al.* 1995). Those studies screened for degrons that exhibited a specific E2 or E3 dependency or filtered out strong degrons. Unlike these previous screens, the present screen did not impose any such filters; we simply asked for defective growth on –Ura medium, anticipating that poor growth would reflect decreased protein half-life. Accordingly, we were successful in isolating a larger number of degrons, for a wider variety of E3s than previously published screens.

Our degron library greatly expands the number of Doa10-dependent degrons available for analysis. Previous studies of Deg1, CL1, and DegAB have proven valuable to begin to dissect the recognition mechanism of Doa10, and led to the suggestion that Doa10 recognizes the hydrophobic face of an amphipathic α-helix present in its substrates (Ravid and Hochstrasser 2008). Substituting hydrophobic residues with polar ones in these degrons was shown to destroy degron function, in some but not all cases (Furth *et al.* 2011; Gilon *et al.* 1998; Johnson *et al.* 1998). However, with only a few examples available up to now, it has remained unclear if all Doa10-dependent degrons must obligatorily contain an amphipathic α-helix, and our understanding of the recognition motif(s) for Doa10 has remained incomplete. Here, we report many new Doa10-dependent degrons. Secondary structure predictions (Bryson *et al.* 2005; Jones 1999) of our sequences (Table S3) suggest that, while some of these conform to the prevailing model, some are predicted to contain α-helices with no clear hydrophobic face, or to contain no helical structure at all. Furthermore, some degrons for which we did not observe any Doa10-dependence, are predicted to form amphipathic helices, suggesting this feature is not sufficient to dictate specificity for Doa10. Our preliminary sequence analysis suggests that a much closer bioinformatic examination, along with mutagenesis experiments to determine minimal length and critical residues of Doa10-dependent degrons, will be necessary to more fully understand the general basis for recognition by Doa10.

The degron library characterized in this study will provide a rich resource for genetic, biochemical, and cell biological studies to identify novel E3s and other UPS components involved in CytoQC, as well as to determine sequence features important for substrate recognition in PQC. Growth on –Ura medium and protein stability assays, together with suppressor screens, can be used to genetically identify new PQC components that act on particular degron constructs. We are currently examining the role of candidate PQC gene products for several of our degron constructs, as well as conducting genome-wide suppressor screens to seek new components. In the work reported here, we looked mainly at single gene deletions or conditional mutations to assess growth, steady state protein levels, and protein half-life. However it is possible that the involvement of a particular E3 or chaperone could be masked by functional redundancy. Thus, we are generating strains deleted for multiple E3s, E2s, or chaperones for further testing. Important questions recently raised for CytoQC are the roles of nuclear import, and aggregation. By tagging our degron constructs with GFP, it will be possible to directly assess their localization and aggregation properties in a broad range of mutants, further extending the utility and versatility of the degrons identified in this study.

## Supplementary Material

Supplemental Material
