# Supplementary material for: Degradation Signals for Ubiquitin-Proteasome Dependent Cytosolic Protein Quality Control (CytoQC) in Yeast
Source: G3 (Bethesda). 2016 Apr 26;6(7):1853–66. doi: 10.1534/g3.116.027953 (PMC4938640; doi:10.1534/g3.116.027953)
Supplement: Supplemental Material [file supp_6_7_1853__index.html]

Degradation Signals for Ubiquitin-Proteasome Dependent Cytosolic Protein Quality Control (CytoQC) in Yeast — Supplemental Material 

# Degradation Signals for Ubiquitin-Proteasome Dependent Cytosolic Protein Quality Control (CytoQC) in Yeast

## Supplemental Material for Maurer *et al.*, 2016

**Files in this Data Supplement:**

- File S1 - Supplemental methods. (.pdf, 135 KB)
- Table S1 - Strains used in this study. (.pdf, 65 KB)
- Table S2 - Plasmids used in this study. (.pdf, 41 KB)
- Figure S1 - Growth of Ura3-HA-degron library isolates in WT versus doa10Δ or other E3 ligases implicated in CytoQC. (.pdf, 11 MB)
- Figure S2 - Doa10-dependent degradation of the degron tester set determined by cycloheximide chase analysis. (.pdf, 688 KB)
- Figure S3 - Steady state analysis of Ura3p-HA-degron proteins in the *ltn1*Δ mutant and the *ubr1*Δ *san*1Δ double mutant. (.pdf, 644 KB)
- Figure S4 - A proteasome-independent high MW species accumulates for the degron fusion protein Ura3p-HA-10-43 in the absence of the Ltn1p ubiquitin E3 ligase. (.pdf, 722 KB)
- Table S3 - Amino acid sequences for Degrons CL1, CL1\* and 77 degrons in the degron collection. (.xlsx, 13 KB)
