# Supplementary material for: Degradation Signals for Ubiquitin-Proteasome Dependent Cytosolic Protein Quality Control (CytoQC) in Yeast
Source: G3 (Bethesda). 2016 Apr 26;6(7):1853–66. doi: 10.1534/g3.116.027953 (PMC4938640; doi:10.1534/g3.116.027953)
Supplement: Supplemental Material [file supp_g3.116.027953_FileS1.pdf]

File S1

Media

Standard minimal dropout media (e.g. SC-Ura, SC-Ura-Leu) were prepared as described previously (Michaelis and Herskowitz 1988). For proteasome inhibition with MG132, the medium used for initial growth was SCP (2% w/v dextrose, 0.17% (w/v) yeast nitrogen base, 0.1% (w/v) proline, supplemented with appropriate amino acids). During MG132 treatment, SCPS medium was used, which is SCP containing 0.0006% SDS to facilitate uptake of the inhibitor (Liu et al. 2007).

Plasmids

To generate the N-terminally Myc-tagged Ura3-HA plasmid pSM2801, recombinational cloning was used to place a single *Myc* epitope tag at the amino-terminus of the Ura3-3XHA-degron protein 12-33 (pSM2743). Plasmid pSM2802 which is C-terminally tagged with *Myc* was created by first generating a PCR fragment derived from pSM2743. The PCR fragments were cloned into pSM2697 at the AatII-SmaI sites, creating Ura3-3XHA-degron-Myc fusion proteins.

Plasmid pSM2803, which tests for stop codon read-through, was created as done for pSM2802, except the PCR fragment contained stop codons immediately upstream and downstream of the *Myc* tag.

Plasmid pSM2811, which contains a polylysine pause sequence (12 AAG) and *Myc* epitope tag downstream of *URA3-HA*, was created by cloning duplexed oligonucleotides into the SmaI site of pSM2697. Plasmid pSM2812 is identical to pSM2811, except a stop codon was included immediately upstream of the *Myc* tag.

Systematic growth analysis for degradation dependencies and quantification of growth

To simplify analyses and condense the library to a single 96-well plate, a subset of 77

unique plasmids that conferred *pre1,2*-dependent growth on SC-Leu-Ura was selected and subjected to further quantitative growth analysis (Figure S1). These 77 plasmids, along with plasmids expressing *URA3-HA-CL1* (pSM2288), *URA3-HA-CL1** (pSM2337; CL1* is a CL1 variant with 9 additional C-terminal residues (Metzger 2009), the positive control plasmid (pSM2697), which expresses *URA3-HA*, and the empty vector (pSM173) (six replicates of each control), were re-arrayed in a randomized order into a single 96-well plate. Control plasmids were placed at various positions to control for differential conditions within a single plate; (Figure S1; A-D). Plasmids were transformed in 96-well format into WT and mutant strains and selected on SC-Leu plates. Transformants from each individual transformation were grown overnight in SC-Leu liquid media in 2.2ml 96-deep-well plates with a single 5mm solid glass bead in each well. Cultures of yeast were transferred to 96-well microtiter plates and used to prepare yeast arrays for growth quantification. Source plates were prepared by manually pinning yeast from the overnight SC-Leu culture onto solid SC-Leu media in quadruplicate, and incubated for two days at 30°C. Yeast were then replica pinned from source plates to solid SC-Leu and SC-Leu-Ura media using a manual pin tool and incubated for 24 hours at 30°C. Plates of yeast arrays were imaged using a flat-bed scanner and growth quantified as follows. The areas of the colonies were measured using CellProfiler (Carpenter et al. 2006; Lamprecht et al. 2007; Vokes and Carpenter 2008). Colony sizes were normalized within plates by scaling the area of each colony according to the mean colony area of the positive controls, Ura3 without a degron, and the mean colony area of the negative controls, no Ura3. Normalized colony size was calculated as:

$$A = \frac{A_c - \bar{A}_n}{\bar{A}_p - \bar{A}_n}$$

where A is the normalized colony size, A_c is the raw colony area, $\bar{A}_n$ the mean area of the negative control colonies, and $\bar{A}_p$ is the mean area of the positive control colonies. For each

degron/strain combination, the mean of the normalized colony size was determined from 24 measurements (six replicate plates with four replicate pinnings per plate). The relative growth between each mutant and the WT was calculated as the Log_2 ratio of the mean normalized colony size of mutant to WT for each degron. Relative growth is reported in the heatmaps for significant differences between mutant and WT, assessed by a Mann-Whitney test.

Preparation of cell lysates, SDS-PAGE, western blotting and imaging

Protein extracts were prepared by treating cells with NaOH (277mM final) containing 2-mercaptoethanol (137mM final) for 15 min on ice, followed by the addition of Trichloroacetic acid (TCA) (5.8% final) and incubation for 15-30 min on ice. TCA precipitates were pelleted by centrifugation at 4°C in a microfuge (5 min), and the pellets were gently rinsed with ice-cold acetone and dried. TCA sample buffer (85 mM Tris-HCl pH8, 120 mM DTT, 80 mM Tris base, 8.5 mM EDTA, 14% glycerol, 3.5% SDS, 0.01% bromophenol blue) was added and samples were incubated at 100°C for 5 min. For cycloheximide chase experiments, where protein extracts were prepared in 96-deep-well plates, centrifugation was performed at 3800 RPM (~3500 x g) for 30-45 min, pellets were not rinsed with acetone before being dried, and after addition of TCA sample buffer, samples were incubated at 95 ^{o}C for 12.5 minutes.

Protein samples corresponding to 0.2 OD_{600} units were resolved by SDS-PAGE (either 12% or 4-15% gradient gels), and transferred to PVDF (Immobilon-FL, Millipore). The membrane was blocked in 10% (v/v) western Blocking Reagent (Roche) diluted in TBST (150 mM NaCl, 10 mM Tris-HCl pH 8, 0.05% (v/v) Tween-20), and incubated in primary antibodies diluted in TBST containing 5% western Blocking Reagent for 1-2 hr at room temperature or overnight at 4°C. HA-fusion proteins were detected with mouse or rat anti-HA monoclonal antibodies (Roche) at 1:1000 dilution. Hexokinase was detected with rabbit anti-hexokinase (a generous gift of Dr. Rob Jensen, The Johns Hopkins University) at 1:200,000 dilution. Myc-fusion proteins were detected with mouse anti-Myc antibody (Millipore) at 1:7000 dilution. Alexa

Fluor 488 goat anti-mouse, Alexa Fluor 647 goat anti-rabbit and Cy3-conjugated goat anti-rabbit antibodies were used to detect immune complexes. After the final TBST wash, membranes were rinsed in 100% methanol, dried and imaged using a PharosFX Plus system (Biorad).

For experiments in Figure 7, which required detection of HA-, Myc- and hexokinase proteins, the PVDF membranes were first incubated with mouse anti-Myc antibody, followed by Alexa Fluor 647 anti-mouse IgG. The membrane was rinsed in 100% methanol, dried and re-wet as per the manufacturer directions. The membrane was then probed with rat anti-HA and rabbit anti-hexokinase, which were detected with Alexa Fluor 488 donkey anti-rat IgG (Invitrogen Molecular Probes) and Cy3 goat anti-rabbit antibodies (Jackson Immunoresearch).
