# Supplementary material for: Degradation Signals for Ubiquitin-Proteasome Dependent Cytosolic Protein Quality Control (CytoQC) in Yeast
Source: G3 (Bethesda). 2016 Apr 26;6(7):1853–66. doi: 10.1534/g3.116.027953 (PMC4938640; doi:10.1534/g3.116.027953)
Supplement: Supplemental Material [file supp_g3.116.027953_TableS1.pdf]

Table S1 Strains used in this study

Strain	Alias	Genotype	Reference/Source
SM4333	WCG4a	*MAT***a** *ura3-5 leu2-3,112 his3-11,15 PRE1 PRE2*	Heinemeyer et al
SM4334	WCG4-11/21a	*MAT***a** *ura3-5 leu2-3,112 his3-11,15 pre1 pre2*	Heinemeyer et al
SM4460	BY4741	*MAT***a** *his3 leu2 met15 ura3*	Open Biosystems
SM4819		*MAT***a** *his3 leu2 met15 ura3 ydj1Δ::KanMX*	Open Biosystems
SM4820		*MAT***a** *his3 leu2 met15 ura3 doa10Δ::KanMX*	Open Biosystems
SM5559		*MAT***a** *his3 leu2 met15 ura3 ltn1Δ::KanMX*	Open Biosystems
SM5745		*MAT***a** *his3 leu2 met15 ura3 ubr1Δ::KanMX*	Open Biosystems
SM5746		*MAT***a** *his3 leu2 met15 ura3 san1Δ::KanMX*	Open Biosystems
SM5755	W303a	*MAT***a** *leu2-3,112 his3-11 trp1-1 ura3-1 can1-100 ade2-1*	Prasad & Ng
SM5770	RHY7450	*MAT***a** *his3 leu2 met15 ura3 ubr1Δ::KanMX san1Δ::NatMX*	Heck et al
SM5774	RPY281	*MAT***a** *leu2-3,112 his3-11 trp1-1 ura3-1 can1-100 ade2-1 ssa1::KanMX ssa2::KanMX*	Prasad & Ng
SM5923	RJD3268	*MAT***a** *leu2-3,112 his3-11 trp1-1 ura3-1 can1-100 ade2-1 uba1::KanMX* pRS313-*UBA1*	Ghaboosi & Deshaies
SM5925	RJD3269	*MAT***a** *leu2-3,112 his3-11 trp1-1 ura3-1 can1-100 ade2-1 uba1::KanMX* pRS313-*uba1-204*	Ghaboosi & Deshaies
