# Supplementary material for: Degradation Signals for Ubiquitin-Proteasome Dependent Cytosolic Protein Quality Control (CytoQC) in Yeast
Source: G3 (Bethesda). 2016 Apr 26;6(7):1853–66. doi: 10.1534/g3.116.027953 (PMC4938640; doi:10.1534/g3.116.027953)
Supplement: Supplemental Material [file supp_g3.116.027953_TableS2.pdf]

Table S2 Plasmids used in this study

Plasmid	Alias	Description	Reference/Source
pSM171	pRS313	[CEN *HIS3*]	Sikorski & Hieter, 1989
pSM173	pRS315	[CEN *LEU2*]	Sikorski & Hieter, 1989
pSM2287		[CEN *LEU2* P_{URA3}-*URA3-3HA*]	Metzger et al, 2008
pSM2288		[CEN *LEU2* P_{URA3}-*URA3-3HA-CL1*]	Metzger et al, 2008
pSM2685	pAP2	[CEN *HIS3 HA-RKR1*]	Braun et al, 2007
pSM2659	pAP3	[CEN *HIS3 HA-rkr1*^{C1508A}]	Braun et al, 2007
pSM2697	pMJM15.1	[CEN *LEU2* P_{URA3}-*URA3-3HA*]	This study
pSM2699	pMJM47	[CEN *LEU2* P_{URA3}-*URA3-3HA::10-1*]	This study
pSM2702	pMJM29	[CEN *LEU2* P_{URA3}-*URA3-3HA::10-6*]	This study
pDM2707	pMJM48	[CEN *LEU2* P_{URA3}-*URA3-3HA::10-13*]	This study
pSM2709	pMJM74	[CEN *LEU2* P_{URA3}-*URA3-3HA::10-15*]	This study
pSM2714	pMJM49	[CEN *LEU2* P_{URA3}-*URA3-3HA::10-21*]	This study
pSM2718	pMJM50	[CEN *LEU2* P_{URA3}-*URA3-3HA::10-31*]	This study
pSM2720	pMJM75	[CEN *LEU2* P_{URA3}-*URA3-3HA::10-34*]	This study
pSM2723	pMJM51	[CEN *LEU2* P_{URA3}-*URA3-3HA::10-40*]	This study
pSM2725	pMJM31	[CEN *LEU2* P_{URA3}-*URA3-3HA::10-43*]	This study
pSM2742	pMJM32	[CEN *LEU2* P_{URA3}-*URA3-3HA::12-32*]	This study
pSM2743	pMJM33	[CEN *LEU2* P_{URA3}-*URA3-3HA::12-33*]	This study
pSM2767	pMJM52	[CEN *LEU2* P_{URA3}-*URA3-3HA::12-86*]	This study
pSM2770	pMJM76	[CEN *LEU2* P_{URA3}-*URA3-3HA::12-98*]	This study
pSM2777	pMJM18	[CEN *LEU2* P_{URA3}-*URA3-3HA-stop-myc*]	This study
pSM2801	pMJM53	[CEN *LEU2* P_{URA3}-*myc-URA3-3HA::12-33*]	This study
pSM2802	pMJM55	[CEN *LEU2* P_{URA3}-*URA3-3HA::12-33-myc*]	This study
pSM2803	pMJM56	[CEN *LEU2* P_{URA3}-*URA3-3HA::12-33-stop-myc-stop*]	This study
pSM2804	pMJM57	[CEN *LEU2* P_{URA3}-*myc-URA3-3HA::10-40*]	This study
pSM2805	pMJM59	[CEN *LEU2* P_{URA3}-*URA3-3HA::10-40-myc*]	This study
pSM2806	pMJM60	[CEN *LEU2* P_{URA3}-*URA3-3HA::10-40-stop-myc-stop*]	This study
pSM2811	pMJM70	[CEN *LEU2* P_{URA3}-*URA3-3HA::K12-myc*]	This study
pSM2812	pMJM71	[CEN *LEU2* P_{URA3}-*URA3-3HA::K12-stop-myc*]	This study
