# Supplementary material for: Degradation Signals for Ubiquitin-Proteasome Dependent Cytosolic Protein Quality Control (CytoQC) in Yeast
Source: G3 (Bethesda). 2016 Apr 26;6(7):1853–66. doi: 10.1534/g3.116.027953 (PMC4938640; doi:10.1534/g3.116.027953)
Supplement: Supplemental Material [file supp_g3.116.027953_Figure_S1.pdf]

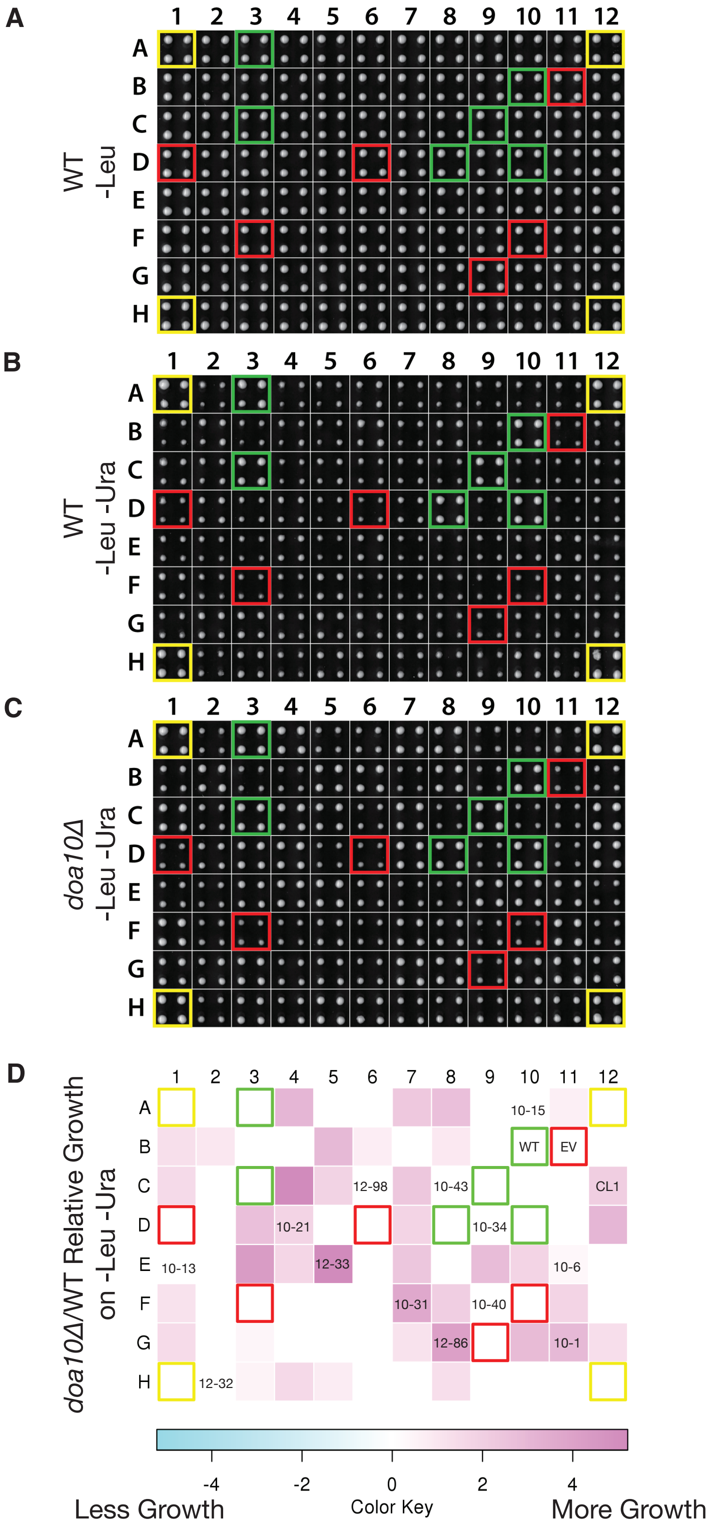
A
B
C
D
1 2 3 4 5 6 7 8 9 10 11 12
A B C D E F G H
WT
-Leu
WT
-Leu -Ura
doa10Δ
-Leu -Ura
doa10Δ/WT Relative Growth
on -Leu -Ura
10-15
WT
EV
12-98
10-43
CL1
10-21
10-34
10-13
12-33
10-6
10-31
10-40
12-86
10-1
12-32
-4
-2
0
2
4
Less Growth
Color Key
More Growth

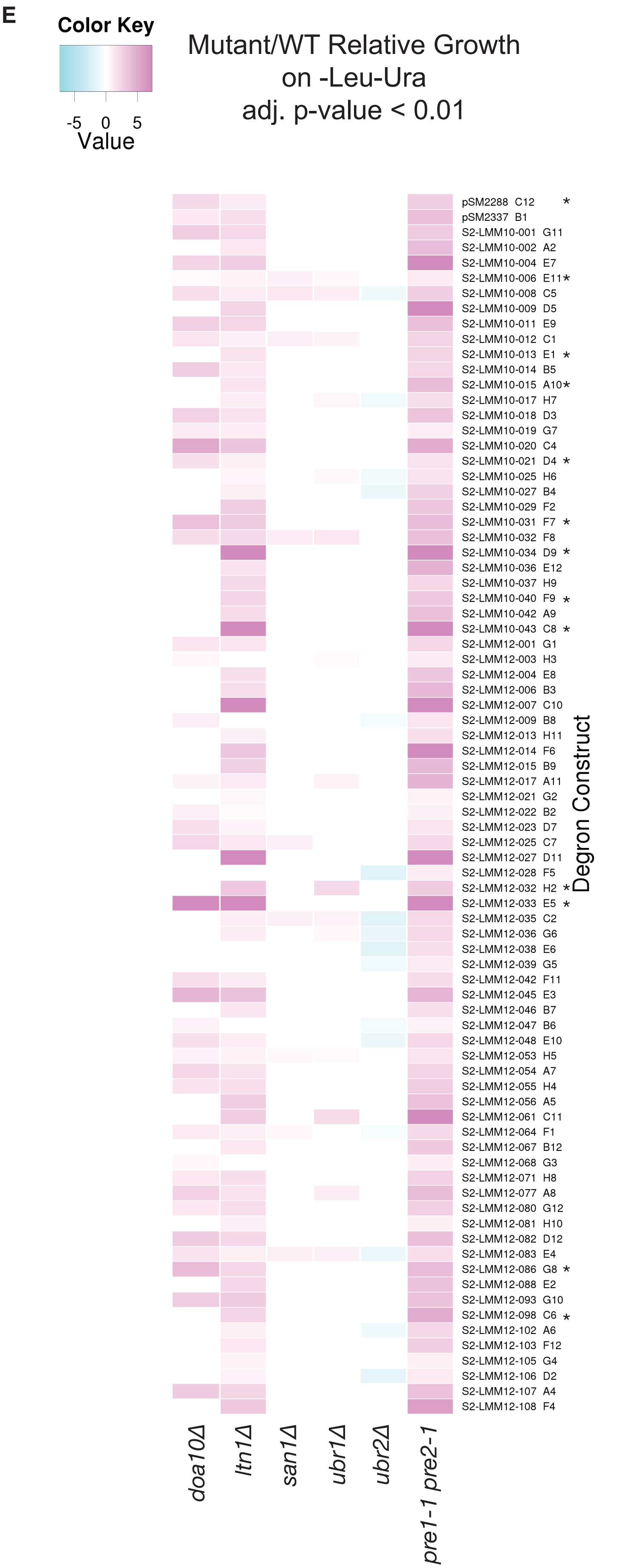
E
Color Key
-5 0 5
Value
Mutant/WT Relative Growth
on -Leu-Ura
adj. p-value < 0.01
pSM2288 C12 *
pSM2337 B1
S2-LMM10-001 G11
S2-LMM10-002 A2
S2-LMM10-004 E7
S2-LMM10-006 E11*
S2-LMM10-008 C5
S2-LMM10-009 D5
S2-LMM10-011 E9
S2-LMM10-012 C1
S2-LMM10-013 E1 *
S2-LMM10-014 B5
S2-LMM10-015 A10*
S2-LMM10-017 H7
S2-LMM10-018 D3
S2-LMM10-019 G7
S2-LMM10-020 C4
S2-LMM10-021 D4 *
S2-LMM10-025 H6
S2-LMM10-027 B4
S2-LMM10-029 F2
S2-LMM10-031 F7 *
S2-LMM10-032 F8
S2-LMM10-034 D9 *
S2-LMM10-036 E12
S2-LMM10-037 H9
S2-LMM10-040 F9 *
S2-LMM10-042 A9
S2-LMM10-043 C8 *
S2-LMM12-001 G1
S2-LMM12-003 H3
S2-LMM12-004 E8
S2-LMM12-006 B3
S2-LMM12-007 C10
S2-LMM12-009 B8
S2-LMM12-013 H11
S2-LMM12-014 F6
S2-LMM12-015 B9
S2-LMM12-017 A11
S2-LMM12-021 G2
S2-LMM12-022 B2
S2-LMM12-023 D7
S2-LMM12-025 C7
S2-LMM12-027 D11
S2-LMM12-028 F5
S2-LMM12-032 H2 *
S2-LMM12-033 E5 *
S2-LMM12-035 C2
S2-LMM12-036 G6
S2-LMM12-038 E6
S2-LMM12-039 G5
S2-LMM12-042 F11
S2-LMM12-045 E3
S2-LMM12-046 B7
S2-LMM12-047 B6
S2-LMM12-048 E10
S2-LMM12-053 H5
S2-LMM12-054 A7
S2-LMM12-055 H4
S2-LMM12-056 A5
S2-LMM12-061 C11
S2-LMM12-064 F1
S2-LMM12-067 B12
S2-LMM12-068 G3
S2-LMM12-071 H8
S2-LMM12-077 A8
S2-LMM12-080 G12
S2-LMM12-081 H10
S2-LMM12-082 D12
S2-LMM12-083 E4
S2-LMM12-086 G8 *
S2-LMM12-088 E2
S2-LMM12-093 G10
S2-LMM12-098 C6 *
S2-LMM12-102 A6
S2-LMM12-103 F12
S2-LMM12-105 G4
S2-LMM12-106 D2
S2-LMM12-107 A4
S2-LMM12-108 F4
Degron Construct
doa10Δ
ltn1Δ
san1Δ
ubr1Δ
ubr2Δ
pre1-1 pre2-1

Figure S1

Figure S1. Growth of Ura3-HA-degron library isolates in WT versus doa10∆ or other E3 ligases implicated in CytoQC. (A-C) Comparing the growth of degron library constructs in WT and *doa10*∆ strains. WT (SM4460) and *doa10Δ* (SM4820) strains transformed with a subset of 77 unique clones isolated in the original degron screen were arrayed in 96-well format, replica-pinned in quadruplicate from solid -Leu media to solid –Leu media (A) and to solid -Leu -Ura media (B, C) and grown for 24 hours. Plates were scanned, and the difference in growth was quantitated and expressed as a heat map (D), in which dark pink, light pink, or white signify a large difference in growth between *doa10*∆ and WT, a small growth difference, or no difference, respectively. Note that Figure S1D is repeated here from Figure 1, for convenience in examining directly together with the actual growth plates. The positions of the 13 representative degrons comprising the "degron tester set" used in later tests are indicated. Controls expressing Ura3p-HA (green boxes), no Ura3p (red boxes), or URA3-HA-CL1 are indicated. Corner positions (yellow boxes) were not used in the analysis. **(E)** Heatmap representation of the relative growth differential for the 77 degron library constructs in strains mutated for the proteasome components Pre1 and Pre2 or E3 ubiquitin ligases known to function in CytoQC, compared to WT strains expressing the same degron. Strains are organized by column, and degrons are organized by rows. Row labels give degron name and its coordinate in 96-well format, corresponding to its location on the growth plates shown in A-D.
