# Supplementary material for: Degradation Signals for Ubiquitin-Proteasome Dependent Cytosolic Protein Quality Control (CytoQC) in Yeast
Source: G3 (Bethesda). 2016 Apr 26;6(7):1853–66. doi: 10.1534/g3.116.027953 (PMC4938640; doi:10.1534/g3.116.027953)
Supplement: Supplemental Material [file supp_g3.116.027953_Figure_S2.pdf]

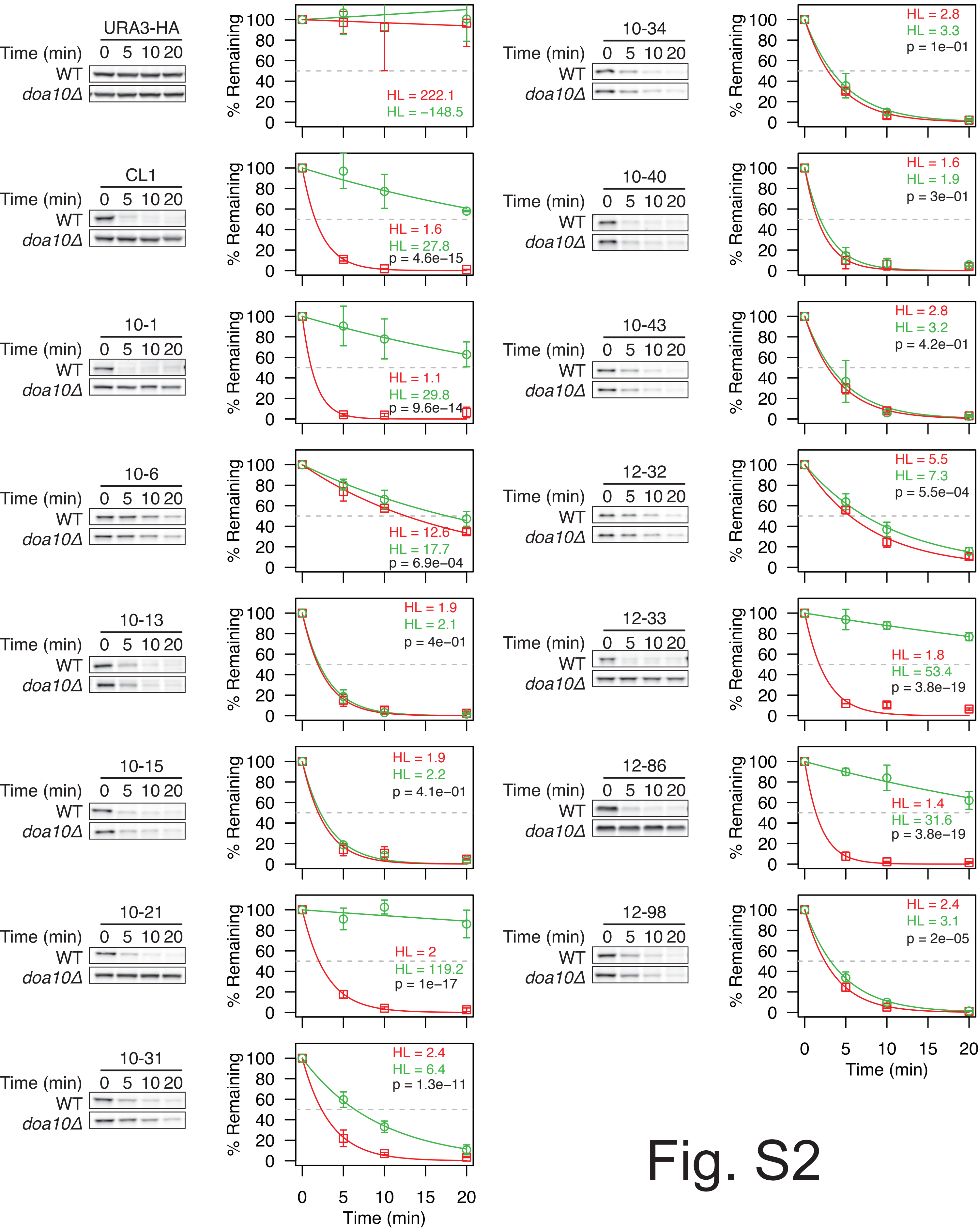
URA3-HA
Time (min) 0 5 10 20
WT
doa10Δ
% Remaining
HL = 222.1
HL = −148.5
CL1
HL = 1.6
HL = 27.8
p = 4.6e−15
10-1
HL = 1.1
HL = 29.8
p = 9.6e−14
10-6
HL = 12.6
HL = 17.7
p = 6.9e−04
10-13
HL = 1.9
HL = 2.1
p = 4e−01
10-15
HL = 1.9
HL = 2.2
p = 4.1e−01
10-21
HL = 2
HL = 119.2
p = 1e−17
10-31
HL = 2.4
HL = 6.4
p = 1.3e−11
10-34
HL = 2.8
HL = 3.3
p = 1e−01
10-40
HL = 1.6
HL = 1.9
p = 3e−01
10-43
HL = 2.8
HL = 3.2
p = 4.2e−01
12-32
HL = 5.5
HL = 7.3
p = 5.5e−04
12-33
HL = 1.8
HL = 53.4
p = 3.8e−19
12-86
HL = 1.4
HL = 31.6
p = 3.8e−19
12-98
HL = 2.4
HL = 3.1
p = 2e−05
Time (min)

Fig. S2

Figure S2. Doa10-dependent degradation of the degron tester set determined by cycloheximide chase analysis. Strains used are SM4460 (WT) and SM4820 (*doa10Δ).* western blots from cycloheximide chase analysis of WT and *doa10Δ* expressing the indicated degron tester set constructs, along with Ura3-HA (no degron) and Ura3-HA-CL1 are shown. Cyclohexamide chase, extract preparation, western blotting, and quantitation are described in Materials and Methods. The half-lives shown here are plotted in the strip chart in Figure 2E.
