# Supplementary material for: Degradation Signals for Ubiquitin-Proteasome Dependent Cytosolic Protein Quality Control (CytoQC) in Yeast
Source: G3 (Bethesda). 2016 Apr 26;6(7):1853–66. doi: 10.1534/g3.116.027953 (PMC4938640; doi:10.1534/g3.116.027953)
Supplement: Supplemental Material [file supp_g3.116.027953_Figure_S3.pdf]

Fig. S3

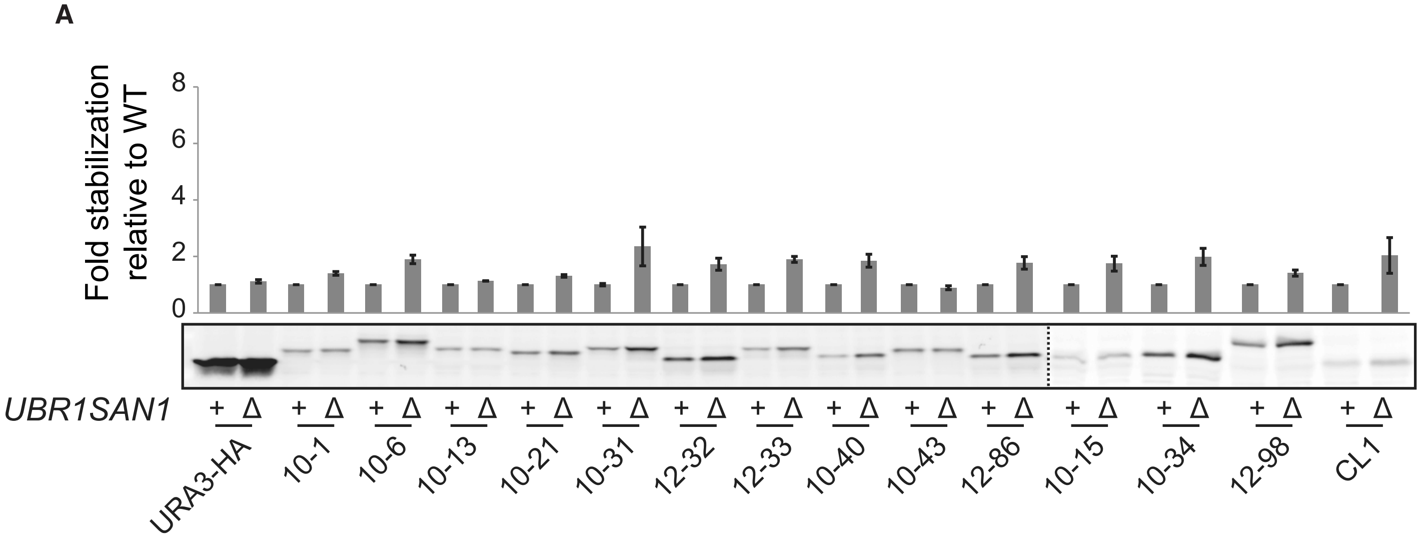
A
Fold stabilization relative to WT
8
6
4
2
0
UBR1SAN1
+ Δ
URA3-HA
10-1
10-6
10-13
10-21
10-31
12-32
12-33
10-40
10-43
12-86
10-15
10-34
12-98
CL1

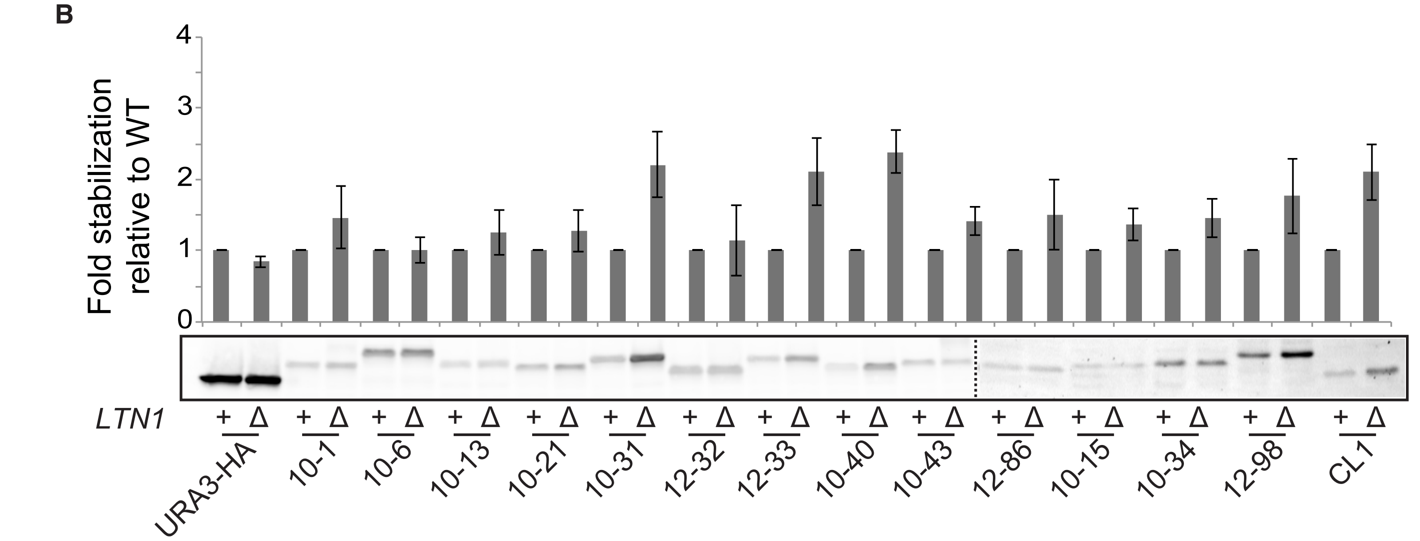
B
Fold stabilization relative to WT
4
3
2
1
0
LTN1
+ Δ
URA3-HA
10-1
10-6
10-13
10-21
10-31
12-32
12-33
10-40
10-43
12-86
10-15
10-34
12-98
CL1

Figure S3. Steady state analysis of Ura3p-HA-degron proteins in the *ltn1*Δ mutant and the *ubr1*Δ *san*1Δ double mutant. Western blots and quantitation of WT (SM4460), *ubr1Δ san1Δ* (SM5770) strains were performed as described in the Materials and Methods. Stabilization, when it occurs, is modest (< 2-fold). Quantitation was performed for three separate experiments, with a representative gel shown here. Vertical dotted lines indicate where separate gels are spliced together.
