# Supplementary material for: Degradation Signals for Ubiquitin-Proteasome Dependent Cytosolic Protein Quality Control (CytoQC) in Yeast
Source: G3 (Bethesda). 2016 Apr 26;6(7):1853–66. doi: 10.1534/g3.116.027953 (PMC4938640; doi:10.1534/g3.116.027953)
Supplement: Supplemental Material [file supp_g3.116.027953_Figure_S4.pdf]

Fig. S4

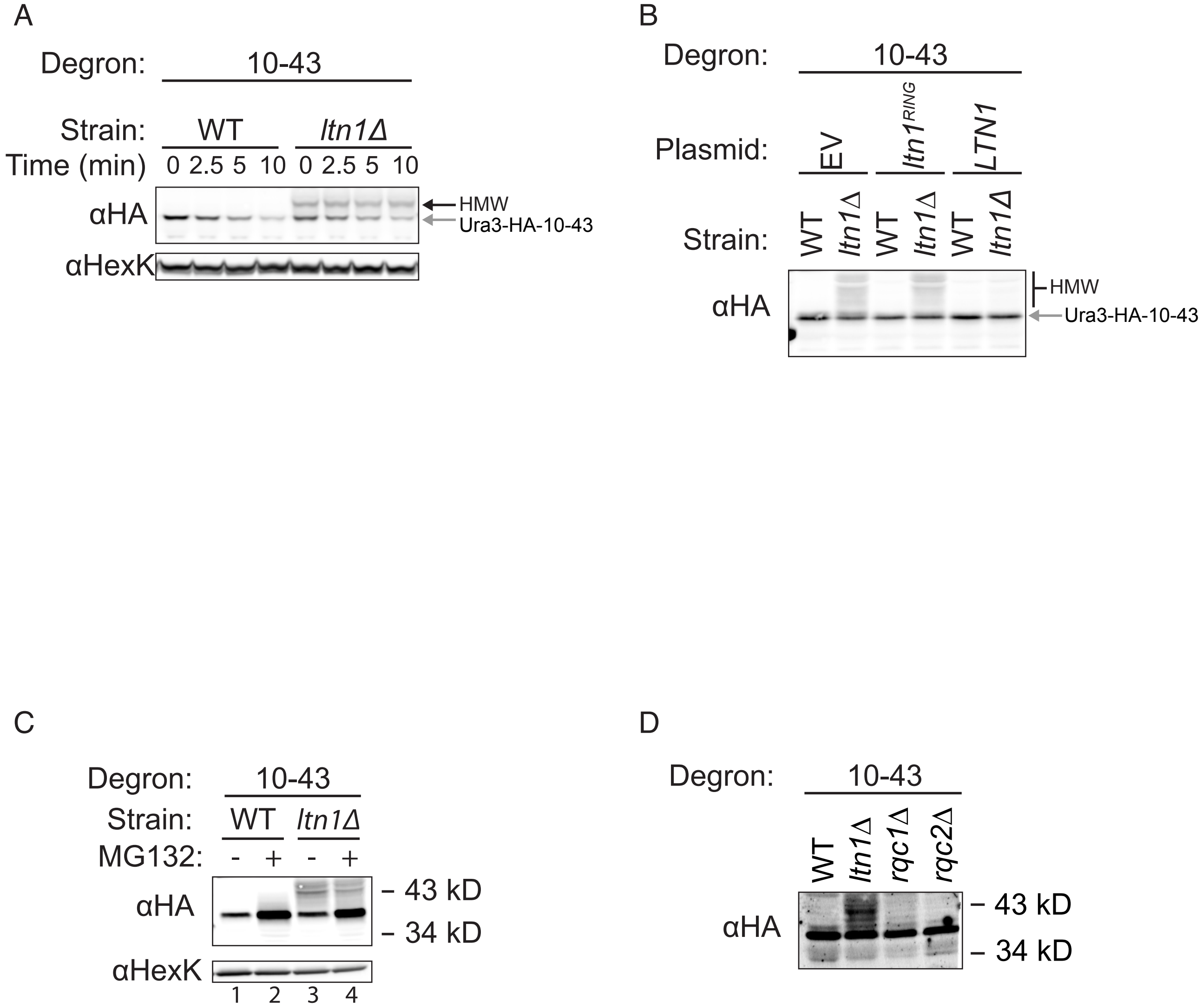
A
Degron:
10-43
Strain:
WT
ltn1Δ
Time (min)
0 2.5 5 10 0 2.5 5 10
αHA
HMW
Ura3-HA-10-43
αHexK
B
Degron:
10-43
Plasmid:
EV
ltn1RING
LTN1
Strain:
WT
ltn1Δ
WT
ltn1Δ
WT
ltn1Δ
αHA
HMW
Ura3-HA-10-43
C
Degron:
10-43
Strain:
WT
ltn1Δ
MG132:
- + - +
αHA
43 kD
34 kD
αHexK
1 2 3 4
D
Degron:
10-43
WT
ltn1Δ
rqc1Δ
rqc2Δ
αHA
43 kD
34 kD

Figure S4. A proteasome-independent high MW species accumulates for the degron fusion protein Ura3p-HA-10-43 in the absence of the Ltn1p ubiquitin E3 ligase. (A) WT (SM4460) and *ltn1*Δ (SM5559) cells expressing Ura3-HA-10-43 (pSM2725) were analyzed by cycloheximide chase analysis as described in Materials and Methods. A high molecular weight (HMW) band or smear is observed only in the *ltn1*Δ strain. **(B)** *ltn1*Δ cells (SM5559) transformed with empty vector (pSM171), plasmid-borne *LTN1* (pSM2658) or $ltn1^{RING}$ mutant (pSM2659) and Ura3-HA-10-43 (pSM2725) were analyzed by western blotting. **(C)** WT (SM4460) and *ltn1*Δ (SM5559) cells expressing Ura3-HA-10-43 (pSM2725) were treated with vehicle (DMSO) or drug (MG132; 75 μM) for two hours prior to immunoblot analysis. **(D)** WT (SM4460), *ltn1*Δ (SM5559), *rqc1*Δ (SM6128) and *rqc2*Δ (SM6129) expressing Ura3-HA-10-43 were analyzed by western blotting.
